# Longitudinal survey of microbiome associated with particulate matter in a megacity

**DOI:** 10.1186/s13059-020-01964-x

**Published:** 2020-03-03

**Authors:** Nan Qin, Peng Liang, Chunyan Wu, Guanqun Wang, Qian Xu, Xiao Xiong, Tingting Wang, Moreno Zolfo, Nicola Segata, Huanlong Qin, Rob Knight, Jack A. Gilbert, Ting F. Zhu

**Affiliations:** 1 Institute of Intestinal Diseases, Department of General Surgery, Shanghai Tenth People’s Hospital, Tongji University School of Medicine, Shanghai, 200072 China; 2 Realbio Genomics Institute, Shanghai, 200050 China; 3 School of Life Sciences, Peking University, Beijing, 100871 China; 4 School of Life Sciences, Tsinghua-Peking Center for Life Sciences, Beijing Frontier Research Center for Biological Structure, Center for Synthetic and Systems Biology, Ministry of Education Key Laboratory of Bioorganic Phosphorus Chemistry and Chemical Biology, Ministry of Education Key Laboratory of Bioinformatics, Tsinghua University, Beijing, 100084 China; 5 Centre for Integrative Biology, University of Trento, 38123 Trento, Italy; 6 Department of Pediatrics, School of Medicine, University of California, San Diego, La Jolla, CA USA; 7 Department of Computer Science and Engineering, University of California, San Diego, La Jolla, CA USA; 8 Center for Microbiome Innovation, University of California, San Diego, La Jolla, CA USA; 9 Scripps Institution of Oceanography, University of California, San Diego, La Jolla, CA 92093 USA

**Keywords:** Particulate matter (PM), Microbiome, Bacteria, Eukaryotes, Viruses, Archaea, Air pollution

## Abstract

**Background:**

While the physical and chemical properties of airborne particulate matter (PM) have been extensively studied, their associated microbiome remains largely unexplored. Here, we performed a longitudinal metagenomic survey of 106 samples of airborne PM_2.5_ and PM_10_ in Beijing over a period of 6 months in 2012 and 2013, including those from several historically severe smog events.

**Results:**

We observed that the microbiome composition and functional potential were conserved between PM_2.5_ and PM_10_, although considerable temporal variations existed. Among the airborne microorganisms, *Propionibacterium acnes*, *Escherichia coli*, *Acinetobacter lwoffii*, *Lactobacillus amylovorus*, and *Lactobacillus reuteri* dominated, along with several viral species. We further identified an extensive repertoire of genes involved in antibiotic resistance and detoxification, including transporters, transpeptidases, and thioredoxins. Sample stratification based on Air Quality Index (AQI) demonstrated that many microbial species, including those associated with human, dog, and mouse feces, exhibit AQI-dependent incidence dynamics. The phylogenetic and functional diversity of air microbiome is comparable to those of soil and water environments, as its composition likely derives from a wide variety of sources.

**Conclusions:**

Airborne particulate matter accommodates rich and dynamic microbial communities, including a range of microbial elements that are associated with potential health consequences.

## Background

As a result of rapid industrialization and urbanization, global megacities have been impacted by extensive and intense particulate matter (PM) pollution events [1], which have potential human health consequences [2–4]. Severe PM pollution is associated with chronic obstructive pulmonary disease (COPD) and asthma, as well as risks for early death [5–8]. While the chemical components of PM pollution and their impacts on human health have been widely studied [9], the potential impact of pollutant-associated microbes remains unclear. Airborne microbial exposure, including exposure to dust-associated organisms, has been established to both protect against and exacerbate certain diseases [10–12]. Understanding the temporal dynamics of the taxonomic and functional diversity of microorganisms in urban air, especially during smog events, will improve our understanding of the potential microbe-associated health consequences. Yet to date, the airborne PM-associated microbiome, especially in urban environments, remains largely understudied [13, 14].

**Fig. 1:**
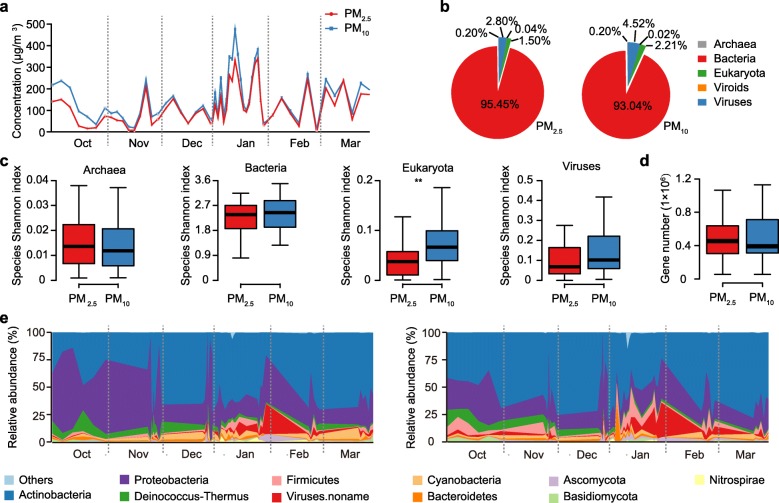
Taxonomic and functional characteristics of air microbiota. **a** Temporal distribution of daily PM concentration variations during the sampling period. **b** Relative abundance of different domains in air microbiome. **c** Taxonomic Shannon index of the PM_2.5_ (red) and PM_10_ (blue) samples. **d** Gene number of the PM_2.5_ (red) and PM_10_ (blue) samples. **e** Temporal distribution of relative abundance from the top 10 most abundant phyla across the sampling period of the PM_2.5_ (left) and PM_10_ (right) samples. Asterisks denote Wilcoxon signed-rank test results; *P* values were adjusted using Benjamini and Hochberg false discovery rate (FDR) (**adjusted *P* < 0.01)

Recent advances in airborne particle DNA extraction and metagenomic library preparation have enabled low biomass environments to be subject to shotgun sequencing analysis [14]. In a previous study, we characterized the microbiome associated with airborne PM in Beijing over a week and identified more than 1300 bacterial, fungal, and viral species [13]. However, this short period is not sufficient to adequately observe the temporal dynamics of this complex microbial system. Thus, we collected 106 airborne PM samples in Beijing between October 2012 and March 2013, during which several record-breaking smog events [15] (Fig. 1a) and high incidences of respiratory diseases [16] occurred, and performed shotgun metagenomic analysis to generate a comprehensive airborne taxonomic and gene catalog, which facilitated the longitudinal characterization of microbial taxonomic diversity and functional potential.

## Results

Based on the annotation by MetaPhlAn2 [17], the overwhelming majority of metagenomic reads from all samples were mapped to bacteria (95.5% of PM_2.5_ and 93.0% of PM_10_), followed by eukaryotes (1.5% of PM_2.5_ and 2.2% of PM_10_), archaea (0.2% of PM_2.5_ and 0.2% of PM_10_), and viruses (2.8% of PM_2.5_ and 4.5% of PM_10_) (Fig. 1b). There was no statistical difference in either the alpha or the beta diversity of species or genes between the PM_2.5_ and PM_10_ samples, with the exception of eukaryotic alpha diversity, which was significantly greater in PM_10_ (Shannon diversity index, Wilcoxon signed-rank test, *P* < 0.01, Fig. 1c, d). The ten bacterial phyla with the greatest proportional representation showed substantial variation throughout the 6-month period, including a significant peak of Firmicutes in PM_10_ samples during the two major smog events in January 2013 (*P* < 0.05; Fig. 1e). In addition, a prominent peak in the proportion of reads annotated to viruses was also found in both PM_2.5_ and PM_10_ samples in late January and February (Fig. 1e, *P* < 0.01).

The metagenomic reads mapped to 702 bacterial species, 27 eukaryotic species, 56 viruses, and 14 archaeal species. The top 50 species of greatest proportion accounted for 71.7% ± 11.8% of the total reads (bacteria 94.6%, eukaryotes 1.4%, viruses 4%) (Additional file 1: Table S3) and exhibited considerable variance in their relative abundance over time, although for the majority of these species, their relative abundance did not significantly differ between PM_2.5_ and PM_10_ (Additional file 2: Figure S1a). Four of the top 50 species, namely *Lactobacillus amylovorus*, *Lactobacillus reuteri*, *Ustilago maydis*, and Porcine type C oncovirus, were at significantly greater proportions in PM_10_ compared with PM_2.5_ (Wilcoxon signed-rank test, adjusted *P* < 0.1). Notably, among the 30 species exhibiting significant differences between the 2 types of samples, 29 were enriched in PM_10_ samples, whereas only 1 was enriched in PM_2.5_ samples (Additional file 1: Table S4, Wilcoxon signed-rank test, adjusted *P* < 0.1). We also correlated distance-corrected dissimilarities of taxonomic community composition with major meteorological factors, of which temperature and dew point had the strongest correlation with taxonomic composition in PM (Additional file 1: Table S5, Additional file 2: Figure S1b). We also monitored the presence of DNA associated with human pathogens in the samples and showed that in both PM_2.5_ and PM_10_, the reads of these hazard microbes displayed only weak correlation with PM concentrations (Additional file 2: Figure S1c); examination of individual pathogen species showed that some peaked in January, coincided with the worst smog event during the study period (Additional file 2: Figure S2). Application of StrainPhlAn analysis revealed considerable strain-level variations in some of the most abundant species, i.e., *Acinetobacter lwoffi*, *Acinetobacter johnsonii*, *Escherichia coli*, *Kocuria* sp. UCR OTCP, *Pantoea ananatis*, *Pantoea dispersa*, *Propionibacterium acnes*, and *Rhodococcus* sp. R04 (Additional file 2: Figure S3-6). Taken together, we observed both similarities and differences in PM_2.5_ and PM_10_ microbiota. PM_10_ or larger particles were formed by aggregation of smaller particles, which could explain the similarities of microbe structure between 2 types of PM samples. The different microbe structures of PM_2.5_ and PM_10_ particles could be a result of the different particulate diameters, since PM_2.5_ has similar sizes as bacteria and PM_10_ has similar sizes as fungi.

We attempted to determine the overlap of core functional genes between the PM pollutants, gut microbiota [18], and ocean microbiota [19] to identify the core functional categories, and compare their relative importance in each database. To this end, we generated a non-redundant gene catalog containing 4,301,891 microbial genes from all PM samples, including 3,278,420 prokaryotic and 1,023,471 eukaryotic genes. The PM core contained considerably reduced functional gene and orthologous group (OG) diversity compared with the human gut and ocean-associated microbiomes, potentially reflecting both the lower biomass and restrictive selective pressures in particulate matter (Kruskal-Wallis rank-sum test, adjusted *P* < 0.05, Additional file 2: Figure S7a-c).

**Fig. 2:**
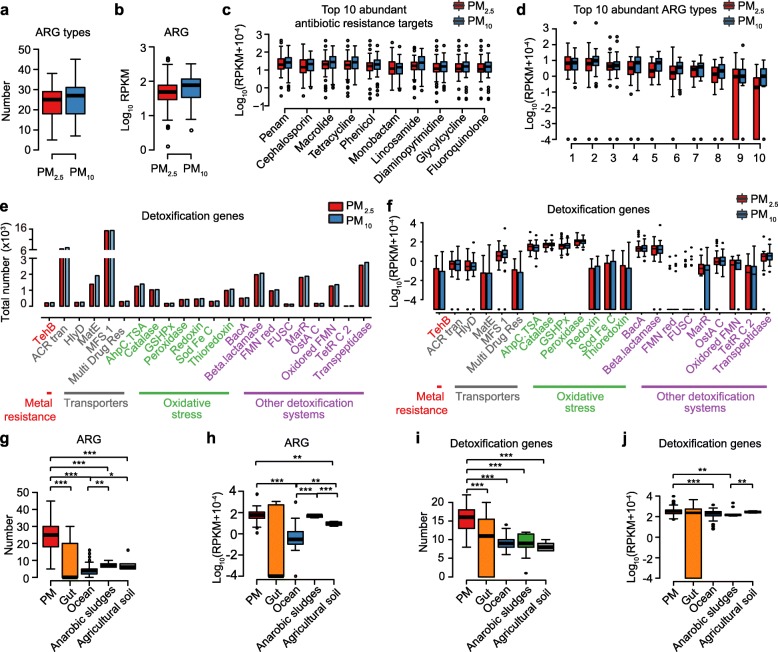
Characteristics of drug resistance and detoxification genes in PM samples. **a** Box plot showing the numbers of antibiotic resistance gene types in PM_2.5_ (red) and PM_10_ (blue) samples. **b** Box plot showing the RPKM values of total antibiotic resistance genes in PM_2.5_ (red) and PM_10_ (blue) samples. **c**, **d** Box plots showing the top 10 most abundant antibiotic resistance targets (**c**) and types (**d**) across PM_2.5_ (red) and PM_10_ (blue) samples. Labels 1–10 represent TEM beta-lactamase, major facilitator superfamily (MFS) antibiotic efflux pump, resistance-nodulation-cell division (RND) antibiotic efflux pump, Erm 23S ribosomal RNA methyltransferase, tetracycline-resistant ribosomal protection protein, lincosamide nucleotidyltransferase (LNU), sulfonamide resistant sul, ABC-F ATP-binding cassette ribosomal protection protein, chloramphenicol acetyltransferase (CAT), and ANT (6), respectively. **e** Bar plot showing the numbers of detoxification genes in PM_2.5_ (red) and PM_10_ (blue) samples. **f** Box plot showing the relative abundance of detoxification genes across PM_2.5_ (red) and PM_10_ (blue) samples. **g**, **h** Box plot showing the number of antibiotic resistance gene types (**g**) and RPKM values of the total antibiotic resistance gene types (**h**) across different environments. **i**, **j** Box plot showing the number of detoxification gene types (**i**) and RPKM values of the total detoxification gene types (**j**) across different environments. Asterisks denote Wilcoxon signed-rank test results; *P* values were adjusted using Benjamini and Hochberg false discovery rate (FDR) (*adjusted *P* < 0.05; **adjusted *P* < 0.01; ***adjusted *P* < 0.001)

Next, genes that potentially confer resistance to 35 different antibiotics were identified. The proportion of genes encoding antibiotic resistance remained stable throughout the study period, and both the number and reads per kilobase per million reads (RPKM) value of antibiotic resistance genes (ARGs) showed no significant differences between PM_2.5_ and PM_10_ samples (Fig. 2a, b), which is suggestive of universal selective pressure within this environment. Genes encoding penam resistance had the greatest overall proportion across PM_2.5_ and PM_10_ (Fig. 2c), while TEM beta-lactamases were the overall most abundant class (Fig. 2d). In addition, we also found several detoxification genes in PM samples including transporters (*ACR transporter*, *MatE*, *MFS*), transpeptidases, and thioredoxins, with the *MFS 1* gene representing the greatest proportion (Fig. 2e, f). PM samples harbored the greatest number of different ARGs and detoxification genes when compared with marine or human gut samples (adjusted *P* < 0.01), but the corresponding RPKM was on par with that of the human gut (Fig. 2g–j). A published computational pipeline [20] was used to estimate the risk of ARGs in PM samples [20]. The resistance risk score was calculated based on the percentage of ARGs associated with mobile genetic elements (MGEs) (Additional file 1: Table S6). In total, there were 982 (5.0%) contigs with ARGs that contained at least 1 MGE (Additional file 1: Table S7) and 379 (1.9%) that contained multidrug resistance clusters (MDRCs) (Additional file 1: Table S8). Notably, a prominent peak of ARG risk was found in January (Additional file 2: Figure S8) when the smog pollution was the most severe.

We next applied network analysis to examine the microbial structure and co-occurrence patterns in samples with different PM metrics [21–24]. We used parameters including betweenness centrality, closeness centrality, and degree [21] to characterize the microbial structure of the two types of samples. Our analysis showed that the network complexity was associated with a higher betweenness, a lower closeness, and a higher degree and was significantly greater in PM_10_ (Additional file 2: Figure S7d, e, adjusted *P* < 0.05). As such, PM_10_ had a denser network, suggesting that a greater number of taxa had similar distributions over the 6-month period, and hence a greater degree of co-association, when compared with the taxa associated with PM_2.5_ samples.

**Fig. 3:**
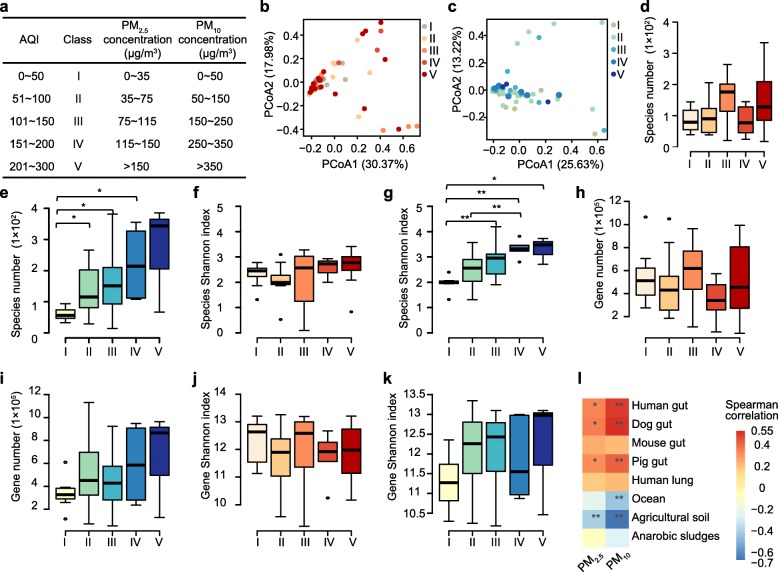
Comparative analysis for five different classes of PM_2.5_ and PM_10_ samples. **a** Stratification of classes I–V for PM_2.5_ and PM_10_ samples. **b**, **c** PCoA analysis based on the Bray-Curtis distance metric of species abundance in PM_2.5_ (**b**) and PM_10_ (**c**) samples. **d**–**g** Taxonomic species number (**d**, **e**) and taxonomic Shannon index (**f**, **g**) for PM_2.5_ (**d**, **f** = red) and PM_10_ (**e**, **g** = blue) samples, respectively. **h**–**k** Gene number (**h**, **i**) and gene Shannon index (**j**, **k**) for PM_2.5_ (red) and PM_10_ (blue) samples, respectively. Asterisks denote Wilcoxon rank-sum test results (**P < 0.05; ***P < 0.01). **l** Pairwise Spearman’s correlation matrix of the portion of airborne microorganisms associated with different environmental sources correlating with PM concentrations (*adjusted *P* < 0.05; **adjusted *P* < 0.01)

We divided the PM_2.5_ and PM_10_ samples into five different classes according to the Air Quality Index (AQI) classification, between which both taxonomic and gene diversity differed (Fig. 3). The PM_10_ samples showed less taxonomic diversity in AQI group I than in AQI groups III and IV (Fig. 3d–k, adjusted *P* < 0.05). The number of detoxification genes was greater in AQI group II than in AQI group I (Additional file 2: Figure S9, adjusted *P* < 0.05). We also analyzed the incidence (per sample detection rate) dynamics of individual species in the five AQI groups. Our analysis revealed that the microbes could be divided into four clusters based on their incidence patterns (Additional file 1: Table S9, Additional file 2: Figure S10a). Cluster 1 comprised more than half of the identified species and was typically identified in less than 10% of the PM samples. Clusters 2 and 3 were depauperate in AQI group I and showed a noticeable increase in incidence in AQI groups II–V. The incidence was mostly below 0.5 except in group V, whereas in cluster 3, the incidence was mostly above 0.5 in all groups. Lastly, species in cluster 4, which was the smallest among the four clusters, maintained a high incidence close to 1 in all five AQI groups of PM particles. The taxonomic compositions of the four clusters differed considerably at both phylum and species levels (Additional file 2: Figure S10b, c, S11).

Subsequently, we calculated the correlation coefficients between individual microbial species and pollutant concentration using MaAsLin [25]. In total, 152 species in PM_10_ samples exhibited a correlation with pollutant concentration, considerably more than that in PM_2.5_ samples (49 species; adjusted *P* < 0.1, Additional file 1: Tables 10 and 11). More importantly, although most of the species identified in PM_10_ samples displayed significant differences in relative abundance between a low AQI level (I or II) and a high AQI level (IV or V), none in PM_2.5_ exhibited such patterns (Wilcoxon rank-sum test, adjusted *P* < 0.1, Additional file 1: Tables 12). The microbes included those associated with human infections, such as *Pseudomonas aeruginosa* [26, 27], with a positive correlation with pollutant concentration for both PM_2.5_ and PM_10_ samples (adjusted *P* < 0.05), and *Stenotrophomonas maltophilia* [28], with a positive correlation with the pollutant concentration in PM_10_ samples. In addition, 72 microbes manifested prominent peaks in January (Additional file 2: Figure S12) when the air pollution was most severe. These included many human commensals and potential human pathogenic agents such as *P. aeruginosa*, *S. maltophilia*, and *Talaromyces marneffei* [29], as well as potential chicken-associated pathogens such as Gallid herpesvirus [30] and avian endogenous retrovirus (AEV) [31].

Finally, using existing metagenomic databases of human, dog, pig, and mouse feces [18, 32–34], we examined the association between potential source of the different microorganisms and the five AQI groups. As pollutant concentrations increased, the proportion of human, dog, and pig feces-associated microbial species significantly increased in both PM_2.5_ samples (*r* = 0.36, 0.37, 0.33, respectively, adjusted *P* < 0.05) and PM_10_ samples (*r* = 0.50, 0.54, 0.43, respectively, adjusted *P* < 0.01), but the trends were more pronounced in PM_10_ samples (Fig. 3l). The inventory of the human feces-associated microbes in PM_10_ samples was also significantly different compared with that in PM_2.5_ samples (*P* = 0.029, ANOSIM test based Bray-Curtis distance metric of species abundance).

## Discussion

Our work revealed a great diversity of microbial species and ARGs in Beijing’s particulate matter, largely consistent with a recent study [35]. The data suggest that potential pathogen and antibiotic resistance burden increases with increasing pollution levels and that severe smog events promote the exposure. In addition, the particulate matter also contained several bacteria that harbored ARGs flanked by mobile genetic elements (Additional file 1: Table S7), which could be associated with horizontal gene transfer. Many of these bacteria were typical or putative members of the human microbiome. Analysis based on AQI groups showed that microbial species exhibited a wide range of incidence and many, including some mammalian gut species, displayed apparent changes in the five AQI groups of samples, and such AQI-related dynamics was affected by the size of particulate matter.

These findings will improve our understanding of microbial dynamics associated with different particulate matter size classes, especially as it pertains to different pollution events. It is possible that the diversity of microorganisms associated with particulate matter results from the reduction in certain environmental stresses such as UV light and desiccation. However, particulate matter also facilitates transient microbial interactions and likely supports substantial levels of extracellular DNA and dead microbes, which may complicate the interpretation of these dynamics.

The potential for particulate matter-associated microbes and viruses to influence human health requires further investigations. Microbial pathogenic transmission through the air, and consequently the risk for infection, can be quantified using metagenomics, as it has been for nosocomial source tracking in hospitals [36, 37], but only if specific infections can be identified in the human population at risk. Any spurious statements about the “presence” of DNA signatures associated with potential pathogens in an environment should be treated as only exploratory evidence of potential risks. Unless these trends can be directly associated with actual infection events in humans or animals, they will remain invalid assessment of health risk. However, routine monitoring and quantification of microbial signatures in atmospheric systems associated with urban environments offer the potential for future retrospective examination of risk events that could promote mitigation strategies. For example, if an outbreak of a multidrug-resistant pathogen infection occurs in a city, being able to determine the environmental and pollution events that are associated with increased risks of exposure could be used as evidence to influence urban policy to reduce exposure. Therefore, the validity of these techniques for continual airborne microbial risk assessment should be further explored, especially for areas of well-understood risks, such as in regions with intensive poultry or pig farming, which could represent zoonotic reservoirs for infection risks.

## Conclusions

Our work provides further evidence for potential environmental and mammalian sources of microbes associated with urban airborne particulate matter and demonstrates differences between pollution levels that could be associated with potential health risks.

## Methods

### Particulate matter and meteorological data collection

The methods used for particulate matter collection were previously described [13, 14]. PM samples were collected from samplers located on the rooftop of a building at Tsinghua University. Three high-volume air samplers (Thermo Electron Corp., MA, USA) were used in this study, with each drawing ambient air at a flow rate of 1.13 m^3^/min for 23 h per day. Two of the samplers were equipped with PM_2.5_ fractionating inlets and one equipped with PM_10_ fractionating inlet. Particulate matters were trapped onto 20.32 × 25.4 cm^2^ Tissuquartz filters (PALL, NY, USA) with 99.9% typical aerosol retention. All of the filters were sterilized by baking in a muffle furnace at 500 °C for 5 h prior to sampling. Before and after deployment in the filter cartridge, each sterilized filter was packaged in a sterilized aluminum foil and stored in a sealed bag. The filter holder and all the tools used for changing new filters were thoroughly cleaned with 75% ethanol or autoclaved every day to avoid contamination. The net weight of each filter was recorded at milligram accuracy before and after sampling, and the weight differences were used to calculate the PM concentration. The filters were stored in aluminum foils and plastic bags at − 80 °C until DNA extraction. The meteorological data were retrieved from the website (https://www.wunderground.com/), using the weather parameters from Beijing Capital Airport (40.07° N, 116.59° E, index number 54511). The weather parameters were exported in a CSV form to be used in further analysis (Additional file 1: Table S1). Tissuquartz filters without exposure to open air were used as negative controls.

### DNA extraction, library construction, and sequencing

We have previously developed an optimized protocol to improve the DNA yield and quality from air particle samples [13, 14]. In this study, we used this technique to extract DNA from PM_2.5_ and PM_10_ samples: 1/4 of PM_10_ filter (a total of ca. 103.04 cm^2^) and 1.5 of the PM_2.5_ filters (a total of ca. 618.24 cm^2^) were used to extract microbial DNA. The filters were cut into 8.96 × 11.5 cm^2^ pieces and placed in 50-mL centrifuge tubes. Next, the filter was soaked into ca. 50 mL 1× PBS buffer. The tubes were pelleted at 4 °C by centrifuging at 200*g* for 3 h. After vortexing, the resuspension was filtered by a 0.2-μm Supor 200 PES Membrane Disc Filter (PALL, NY, USA). The PALL filter was then used as the starting material for pretreatment by MO-BIO PowerSoil DNA isolation kit (Carlsbad, CA, USA). The samples were then incubated at 65 °C in PowerBead Tubes for 15 min followed by vortexing for 10 min. The following steps were performed according to the standard MO-BIO PowerSoil DNA isolation protocol except for the column purification step, which was replaced with magnetic bead purification (Agencourt AMPure XP, Beckman, CA, USA) for improved DNA yield. Extracted DNA samples were diluted in 50 μL sterilized water. We used the Qubit 2.0 fluorometer (Thermo Fisher Scientific Inc. MA, USA) to quantify the concentration of DNA. Library preparation was performed according to the manufacturer’s instructions (Illumina, CA, USA). We constructed a paired-end library with an insert size of 500 bp. A starting amount of 5 ng DNA from each DNA sample was used for library preparation in order to ensure sample consistency. In order to minimize possible bias introduced by PCR, 12 cycles were performed during PCR amplification.

### Metagenomic sequencing

The quality of all DNA libraries was evaluated using an Agilent bioanalyzer (Agilent Technologies, CA, USA) with the DNA LabChip 1000 kit. Whole-genome shotgun sequencing of PM samples collected was carried out on the Illumina Hiseq 4000 platform (Illumina, CA, USA) with 150-bp paired-end read length. In total, we obtained 946-Gb raw data (mean 8.8 Gb per sample, mean insert size 354 ± 83 bp). The raw reads of metagenomic sequencing were processed to remove low-quality reads and adaptor contaminations. Bases with a quality score < 30 were trimmed from 3′ end of reads, and reads < 70 bp were removed. Finally, we obtained 882-Gb clean data (mean 8.2 Gb per sample), and the proportion of high-quality reads was about 92.12% on average in all samples.

### De novo assembly and gene catalog construction

De novo assembly of clean reads was performed using MetaVelvet-SL (version 1.2.02) [38] with 63-kmer. Furthermore, the gaps in the scaffold were filled using GapCloser [39]. A total of 3.58 million contigs were generated (minimum length of 300 bp). These contigs had a total length of 4.97 Gb and an average N50 length of 12,243 bp and ranged from 669 to 62,034 bp (Additional file 1: Table S2). To create a multi-kingdom gene catalog of inhalable airborne microorganisms in Beijing’s PM_2.5_ and PM_10_ pollutants, two gene prediction methods were performed, namely, MetaGeneMark [40] (version 3.26) for prokaryotic microorganisms (bacteria, archaea, and virus) and Augustus [41] for eukaryotic microorganisms (fungi). For contigs that both prokaryotic and eukaryotic genes were called, we used the Taxator-tK [42] to assign them to a kingdom and the corresponding predicted genes were reserved. Contigs that were not identified by Taxator-tK were removed, accounting for 10% in length of all contigs which harbor genes. Contigs assigned to animals or plants by Taxator-tK were removed. A non-redundant gene catalog was constructed using CD-HIT [43] (version 4.5.7) with a sequence identity cutoff of 95% and a minimum coverage cutoff of 90% for shorter sequences. The final non-redundant gene catalog contains 4,301,891 microbial genes, including 3,278,420 prokaryotic genes and 1,023,471 eukaryotic genes. We compared the core genes of PM pollutants to gut microbiota and ocean microbiota using the criteria of 95% sequence identity and 90% alignment coverage of the shorter sequence.

### Taxonomic and functional profiling

Metagenomic reads were taxonomically profiled using MetaPhlAn2 with default parameter settings. The metagenomic gene catalog was annotated by alignment against the proteins in the eggNOG 3.0 database [44] and KEGG database [45] using BLASTP (*E* value ≤ 1E−5). A gene was assigned to an OG or KO by the highest scoring annotated hit with at least one HSP (high-scoring segment pair) scoring > 60. For query genes with multiple matches, the annotated reference gene with the highest score was used. For each functional feature (OG in eggNOG or KO in the KEGG database), we estimated its abundance by accumulating the relative abundance of all genes from belonging to the same family. HUMAnN2 [46] was subsequently used to calculate the relative abundance of metabolic pathways in the MetaCyc database [47]. Potential pathogens were identified by first searching the MetaPhlAn-annotated species in Microbial Genome Database System (http://data.mypathogen.org/search/genomeSearch) for human pathogens. The resulting microbes were further validated by PubMed search to ensure that each had been reported in human infection or human diseases.

### Strain-level analysis

Strain-level profiling was performed with StrainPhlAn. For each sample, the clean reads were first mapped against the MetaPhlAn2 markers by Bowtie2 [48] and then the consensus sequences were produced according to the mapping result. The consensus sequences represent the most abundant strains for each species in a sample. Similarly, the consensus sequences of public reference genomes of stains for each species were obtained by aligning the markers to these genomes. Finally, the extracted consensus sequences of references and samples were multiply aligned by MUSCLE [49], and the phylogenetic trees were built by RAxML [50] (parameters: -m GTRCAT and -p 1234).

### Antibiotic resistance and detoxification genes

Predicted genes were annotated with BLASTX (*E* value < 1E−10, identity > 60%, and minimum alignment length > 25 amino acids) against the CARD database [51]. Multidrug resistance clusters were identified as contigs containing multiple antibiotic resistance proteins. Employing the method described in the previous paper [52], the Gene Ontology annotation [46] and Pfam (release 24) [53] were used to create the detoxification protein database, which contains 31 strictly detoxification-related protein families with profile HMMs. All gene sequences were scanned against the database of profile HMMs using hmmsearch, a part of the HMMER3 software [54]. ShortBRED [55] was used to quantify the abundance of antibiotic resistance genes and detoxification genes. ShortBRED markers were identified from the annotated antibiotic resistance proteins or detoxification proteins using the reference database of Swiss-Prot in Uniprot. Clean reads were mapped against these marker sequences with 99% sequence identity. All analyses were performed on gene abundance normalized to reads per kilobase per million reads (RPKM).

### Mobile genetic elements

Putative MGEs were identified from the contigs with BLASTN (*E* value < 1E−10, identity > 60%, coverage of the MGE reference > 90%) against the ACLAME database, comprising known plasmids, bacteriophages, and transposons [56].

### Co-occurrence network

To construct the meta-community co-occurrence network, we first removed species with relative abundances less than 0.01% or present in less than 10 samples. The Spearman correlation coefficients between species were computed using cor.test function in R 3.6.2 (Lucent Technologies, NJ, USA), and all the *P* values were adjusted for multiple testing using the Benjamini and Hochberg false discovery rate (FDR) controlling procedure. Based on correlation coefficients (> 0.78) and FDR (< 0.05) adjusted *P* values for correlation, we constructed the co-occurrence network. The cutoff of correlation coefficients was determined as 0.78 through random matrix theory-based methods [57]. Network properties were calculated with the igraph package. The co-occurrence networks were visualized by Gephi.

### Statistical analysis

Adjustment for multiple testing was performed using Benjamini and Hochberg false discovery rate (FDR) controlling procedure (p.adjust function in R). To explain the Bray-Cutis distance of taxonomic community composition with major meteorological factors, the permutational multivariate analysis of variance was employed using the function adonis from the R package vegan. The *P* value was determined by 999 permutations and was subsequently adjusted by the Benjamini and Hochberg method.

## Supplementary information

**Additional file 1: Table S1.** Summary of PM and meteorological data. **Table S2.** Summary of sequencing and assembly data. **Table S3.** Top 50 abundant species in PM samples. **Table S4.** Significant putative genus/species between PM_2.5_ and PM_10_ samples identified by Wilcoxon signed-rank test (FDR <0.1). **Table S5.** PERMANOVA for the influence of Meteorological factors on the taxonomic profile. **Table S6.** Antibiotic resistome risk analysis of PM samples. **Table S7.** Antibiotic resistance contigs contained at least one MGEs. **Table S8.** Antibiotic resistance contigs contained multidrug resistance clusters. **Table S9.** Species clusters based on their incidence patterns in the five PM concentration levels of PM_10_. **Table S10.** Associations of PM_10_ concentration with individual species (FDR <0.05). **Table S11.** Associations of PM_2.5_ concentration with individual species (FDR <0.05). **Table S12.** Significant putative species identified by Wilcoxon rank-sum test between different PM_10_ concentration levels (FDR <0.1).

**Additional file 2: Figure S1.** Most abundant microorganism species identified from airborne particulate matters (a) and the meteorological factors associated with PM microbiome (b). a, Box plot of the daily variations of the relative abundance of the top 50 most abundant microorganism species in PM samples. Boxes correspond to the interquartile range between the 25th and 75th percentiles, and the central lines represent the 50th percentile. Ends of the central lines correspond to the lowest and highest values no more than 1.5 times the interquartile range from the box, while circles represent the outliers. Red, PM_2.5_; Blue, PM_10_. b, The bar plot shows the explained variation of each factor in the variation of microbial composition [Bray–Curtis (BC) distance]. c. Spearman rank-order correlation plot showing the relationship between pathogen mapped reads (%) and PM concentration. **Figure S2.** Temporal distribution of the daily relative abundance of 96 human pathogens and PM concentration variations during the sampling time. **Figure S3.** Strain-level phylogenetic trees of *Escherichia coli*. Black, reference strains; red, MetaSUB samples; green, PM samples. **Figure S4.** Strain-level phylogenetic trees of *Propionibacterium acnes* (a), *Acinetobacter lwoffi* (b) *and Pantoea ananatis* (c)*.* Black, reference strains; red, MetaSUB samples; green, PM samples. **Figure S5.** Strain-level phylogenetic trees of *Kocuria* sp. UCD OTCP (a), *Acinetobacter johnsonii* (b) and *Pantoea dispersa* (c). Black, reference strains; red, MetaSUB samples; green, PM samples. **Figure S6.** Strain-level phylogenetic trees of *Rhodococcus* sp. R04. Black, reference strains; red, MetaSUB samples; green, PM samples. **Figure S7.** Comparison of genesets from PM, ocean and gut microbiota (a, b, c) and the network topological variables of PM microbiota (d, e). a, Venn diagram indicating a low overlap of PM, human gut and ocean gene catalog. b, Venn diagram of core OGs suggesting a large overlap of functions among PM, human gut and ocean microbiota. c, Bar chart showing the comparison of gene abundance summarized into OG functional categories. A, RNA processing and modification; B, Chromatin structure and dynamics; C, Energy production and conversion; D, Cell cycle control, cell division, chromosome partitioning; E, Amino acid transport and metabolism; F, Nucleotide transport and metabolism; G, Carbohydrate transport and metabolism; H, Coenzyme transport and metabolism; I, Lipid transport and metabolism; J, Translation, ribosomal structure and biogenesis; K, Transcription; L, Replication, recombination and repair; M, Cell wall/membrane/envelope biogenesis; N, Cell motility; O, Posttranslational modification, protein turnover, chaperones; P, Inorganic ion transport and metabolism; Q, Secondary metabolites biosynthesis, transport and catabolism; R, General function prediction only; S, Function unknown; T, Signal transduction mechanisms; U, Intracellular trafficking, secretion, and vesicular transport; V, Defense mechanisms; W, Extracellular structures; Z, Cytoskeleton. d, The distribution of degree of nodes in the networks. e, Network topological variables comparison between PM_2.5_ (red) and PM_10_ (blue) samples (up), PM_2.5_ <75 μg/m^3^ and PM_2.5_ >75 μg/m^3^ (middle), as well as PM_10_ <150 μg/m^3^ and PM_10_ >150 μg/m^3^ (bottom). Asterisks denote Kruskal-Wallis test results, p-values were adjusted using Benjamini and Hochberg false discovery rate (FDR) (*, adjusted *P* <0.05, **, adjusted *P* <0.01, ***, adjusted *P* <0.001). **Figure S8.** Temporal distribution of daily resistance risk and PM concentration variations during the sampling time. (a-e) The percentage of contigs with ARG (a), MGE (b), pathogen (c), ARG&MGE (d) and ARG&MGE&pathogen (e) in all contigs. (f) The resistance risk score. **Figure S9.** Comparative analysis of antibiotic resistance and detoxification gene for 5 different classes of PM_2.5_ and PM_10_ samples. a, b, c, d, show the numbers of antibiotic resistance gene types (a, b) and RPKM values of the total antibiotic resistance gene types (c, d) in PM_2.5_ (red) and PM_10_ (blue) samples, respectively. e, f, g, h, show the numbers of detoxification gene types (e, f) and RPKM values of the total detoxification gene types (g, h) in PM_2.5_ (red) and PM_10_ (blue) samples, respectively. Asterisks denote Kruskal-Wallis test results, p-values were adjusted using Benjamini and Hochberg false discovery rate (FDR) (*, adjusted *P* <0.05). **Figure S10.** Species clusters based on their incidence patterns in the five PM concentration levels of PM_10_ samples. a, Hierarchical Ward-linkage clustering of species based on their incidence patterns in the five PM concentration levels of PM_10_ samples. Colouring represents the incidence (per sample detection rate). b, Proportion of species affiliating to each of the 5 phyla in the four species clusters of PM_10_ samples. c, The heat map of relative abundance of species in cluster 4. Colouring represents the relative abundance of species. **Figure S11.** Heat map of relative abundance of species in cluster 2 (a) and cluster 3 (b). **Figure S12.** Temporal distribution of the daily relative abundance of 72 microbes and PM concentration variations during the sampling time.

**Additional file 3.** Review history.

## Data Availability

Sequencing data are available at NCBI under the bioproject number PRJNA486429 [58].

## References

[1] Zhang RY, Wang GH, Guo S, Zarnora ML, Ying Q, Lin Y, Wang WG, Hu M, Wang Y (2015). Formation of urban fine particulate matter. Chem Rev.

[2] Zhang Q, He K, Huo H (2012). Policy: cleaning China’s air. Nature.

[3] Lee JY, Park EH, Lee S, Ko G, Honda Y, Hashizume M, Deng F, Yi S-M, Kim H (2017). Airborne bacterial communities in three east asian cities of China, South Korea, and Japan. Sci Rep.

[4] Cleaner urban air tomorrow? [Editorial]. Nature Geosci. 2017;10:69.

[5] Kim K-H, Kabir E, Kabir S (2015). A review on the human health impact of airborne particulate matter. Environ Int.

[6] Zheng S, Pozzer A, Cao C, Lelieveld J. Long-term (2001–2012) concentrations of fine particulate matter (PM_2.5_) and the impact on human health in Beijing, China. Atmos Chem Phys. 2015;15:5715–25.

[7] Walton H, Dajnak D, Beevers S, Williams M, Watkiss P, Hunt A (2015). Understanding the health impacts of air pollution in London.

[8] Conibear L, Butt EW, Knote C, Arnold SR, Spracklen DV (2018). Residential energy use emissions dominate health impacts from exposure to ambient particulate matter in India. Nat Commun.

[9] Huang R-J, Zhang Y, Bozzetti C, Ho K-F, Cao J-J, Han Y, Daellenbach KR, Slowik JG, Platt SM, Canonaco F (2014). High secondary aerosol contribution to particulate pollution during haze events in China. Nature.

[10] Stein MM, Hrusch CL, Gozdz J, Igartua C, Pivniouk V, Murray SE, Ledford JG, Marques dos Santos M, Anderson RL, Metwali N (2016). Innate immunity and asthma risk in Amish and Hutterite farm children. N Engl J Med.

[11] Valkonen M, Täubel M, Pekkanen J, Tischer C, Rintala H, Zock JP, Casas L, Probst-Hensch N, Forsberg B, Holm M (2018). Microbial characteristics in homes of asthmatic and non-asthmatic adults in the ECRHS cohort. Indoor Air.

[12] Bharadwaj P, Zivin JG, Mullins JT, Neidell M (2016). Early-life exposure to the great smog of 1952 and the development of asthma. Am J Respir Crit Care Med.

[13] Cao C, Jiang W, Wang B, Fang J, Lang J, Tian G, Jiang J, Zhu TF. Inhalable microorganisms in Beijing’s PM_2. 5_ and PM_10_ pollutants during a severe smog event. Environ Sci Technol. 2014;48:1499–507. 10.1021/es4048472 PMC3963435 24456276

[14] Jiang W, Liang P, Wang B, Fang J, Lang J, Tian G, Jiang J, Zhu TF (2015). Optimized DNA extraction and metagenomic sequencing of airborne microbial communities. Nat Protoc.

[15] Ouyang Y (2013). China wakes up to the crisis of air pollution. Lancet Respir Med.

[16] Xu Q, Li X, Wang S, Wang C, Huang F, Gao Q, Wu L, Tao L, Guo J, Wang W (2016). Fine particulate air pollution and hospital emergency room visits for respiratory disease in urban areas in Beijing, China, in 2013. PLoS One.

[17] Truong DT, Franzosa EA, Tickle TL, Scholz M, Weingart G, Pasolli E, Tett A, Huttenhower C, Segata N (2016). MetaPhlAn2 for enhanced metagenomic taxonomic profiling (vol 12, pg 902, 2015). Nat Methods.

[18] Wen C, Zheng Z, Shao T, Liu L, Xie Z, Le Chatelier E, He Z, Zhong W, Fan Y, Zhang L (2017). Quantitative metagenomics reveals unique gut microbiome biomarkers in ankylosing spondylitis. Genome Biol.

[19] Sunagawa S, Coelho LP, Chaffron S, Kultima JR, Labadie K, Salazar G, Djahanschiri B, Zeller G, Mende DR, Alberti A (2015). Structure and function of the global ocean microbiome. Science.

[20] Oh M, Pruden A, Chen C, Heath LS, Xia K, Zhang L. MetaCompare: a computational pipeline for prioritizing environmental resistome risk. FEMS Microbiol Ecol. 2018;94:1–9. 10.1093/femsec/fiy079 PMC5995210 29718191

[21] Ma B, Wang H, Dsouza M, Lou J, He Y, Dai Z, Brookes PC, Xu J, Gilbert JA (2016). Geographic patterns of co-occurrence network topological features for soil microbiota at continental scale in eastern China. ISME J.

[22] Ruan Q, Dutta D, Schwalbach MS, Steele JA, Fuhrman JA, Sun F (2006). Local similarity analysis reveals unique associations among marine bacterioplankton species and environmental factors. Bioinformatics.

[23] Fuhrman JA, Steele JA (2008). Community structure of marine bacterioplankton: patterns, networks, and relationships to function. Aquat Microb Ecol.

[24] Faust K, Sathirapongsasuti JF, Izard J, Segata N, Gevers D, Raes J, Huttenhower C (2012). Microbial co-occurrence relationships in the human microbiome. PLoS Comput Biol.

[25] Morgan XC, Tickle TL, Sokol H, Gevers D, Devaney KL, Ward DV, Reyes JA, Shah SA, LeLeiko N, Snapper SB (2012). Dysfunction of the intestinal microbiome in inflammatory bowel disease and treatment. Genome Biol.

[26] Nelson JW, Tredgett MW, Sheehan J, Thornton D, Notman D, Govan J (1990). Mucinophilic and chemotactic properties of Pseudomonas aeruginosa in relation to pulmonary colonization in cystic fibrosis. Infect Immun.

[27] Bacci G, Mengoni A, Fiscarelli E, Segata N, Taccetti G, Dolce D, Paganin P, Morelli P, Tuccio V, De Alessandri A (2017). A different microbiome gene repertoire in the airways of cystic fibrosis patients with severe lung disease. Int J Mol Sci.

[28] Talmaciu I, Varlotta L, Mortensen J, Schidlow DV (2000). Risk factors for emergence of Stenotrophomonas maltophilia in cystic fibrosis. Pediatr Pulmonol.

[29] Le T, Ly VT, Thu NTM, Nguyen A, Thanh NT, Vinh Chau NV, Thwaites G, Perfect J, Kolamunnage-Dona R, Hope W: Population pharmacodynamics of amphotericin B deoxycholate for disseminated infection caused by *Talaromyces marneffei*. Antimicrobial Agents and Chemotherapy 2018:AAC.01739–01718. 10.1128/AAC.01739-18 PMC6355582 30420478

[30] Laursen AMS, Kulkarni RR, Tahaabdelaziz K, Plattner BL, Read LR, Sharif S (2018). Characterizaton of gamma delta T cells in Marek’s disease virus (Gallid herpesvirus 2) infection of chickens. Virology.

[31] Hu X, Zhu W, Chen S, Liu Y, Sun Z, Geng T, Song C, Gao B, Wang X, Qin A (2017). Expression patterns of endogenous avian retrovirus ALVE1 and its response to infection with exogenous avian tumour viruses. Arch Virol.

[32] Coelho LP, Kultima JR, Costea PI, Fournier C, Pan Y, Czarnecki-Maulden G, Hayward MR, Forslund SK, Schmidt TSB, Descombes P (2018). Similarity of the dog and human gut microbiomes in gene content and response to diet. Microbiome.

[33] Xiao L, Estelle J, Kiilerich P, Ramayo-Caldas Y, Xia Z, Feng Q, Liang S, Pedersen AØ, Kjeldsen NJ, Liu C (2016). A reference gene catalogue of the pig gut microbiome. Nat Microbiol.

[34] Xiao L, Feng Q, Liang S, Sonne SB, Xia Z, Qiu X, Li X, Long H, Zhang J, Zhang D (2015). A catalog of the mouse gut metagenome. Nat Biotechnol.

[35] Pal C, Bengtsson-Palme J, Kristiansson E, Larsson DJ (2016). The structure and diversity of human, animal and environmental resistomes. Microbiome.

[36] Pham TM, Kretzschmar M, Bertrand X, Bootsma M, on behalf of C-MC (2019). Tracking Pseudomonas aeruginosa transmissions due to environmental contamination after discharge in ICUs using mathematical models. PLoS Comput Biol.

[37] Reigadas E, Vazquez-Cuesta S, Onori R, Villar-Gomara L, Alcala L, Marin M, Martin A, Munoz P, Bouza E. Clostridioides difficile contamination in the environment of a clinical microbiology laboratory and laboratory workers. Clin Microbiol Infect. 2019;26:340–4. 10.1016/j.cmi.2019.06.027 31284033

[38] Sato K, Sakakibara Y (2014). MetaVelvet-SL: an extension of the Velvet assembler to a de novo metagenomic assembler utilizing supervised learning. DNA Res.

[39] Luo R, Liu B, Xie Y, Li Z, Huang W, Yuan J, He G, Chen Y, Pan Q, Liu Y (2012). SOAPdenovo2: an empirically improved memory-efficient short-read de novo assembler. Gigascience.

[40] Noguchi H, Park J, Takagi T (2006). MetaGene: prokaryotic gene finding from environmental genome shotgun sequences. Nucleic Acids Res.

[41] Stanke M, Schöffmann O, Morgenstern B, Waack S (2006). Gene prediction in eukaryotes with a generalized hidden Markov model that uses hints from external sources. BMC bioinformatics.

[42] Dröge J, Gregor I, McHardy AC (2014). *Taxator-tk*: precise taxonomic assignment of metagenomes by fast approximation of evolutionary neighborhoods. Bioinformatics.

[43] Li W, Godzik A (2006). Cd-hit: a fast program for clustering and comparing large sets of protein or nucleotide sequences. Bioinformatics.

[44] Jensen LJ, Julien P, Kuhn M, von Mering C, Muller J, Doerks T, Bork P (2007). eggNOG: automated construction and annotation of orthologous groups of genes. Nucleic Acids Res.

[45] Kanehisa M, Goto S, Kawashima S, Okuno Y, Hattori M (2004). The KEGG resource for deciphering the genome. Nucleic Acids Res.

[46] Abubucker S, Segata N, Goll J, Schubert AM, Izard J, Cantarel BL, Rodriguez-Mueller B, Zucker J, Thiagarajan M, Henrissat B (2012). Metabolic reconstruction for metagenomic data and its application to the human microbiome. PLoS Comput Biol.

[47] Caspi R, Altman T, Billington R, Dreher K, Foerster H, Fulcher CA, Holland TA, Keseler IM, Kothari A, Kubo A (2013). The MetaCyc database of metabolic pathways and enzymes and the BioCyc collection of Pathway/Genome Databases. Nucleic Acids Res.

[48] Langmead B, Salzberg SL (2012). Fast gapped-read alignment with Bowtie 2. Nat Methods.

[49] Edgar RC (2004). MUSCLE: multiple sequence alignment with high accuracy and high throughput. Nucleic Acids Res.

[50] Stamatakis A (2014). RAxML version 8: a tool for phylogenetic analysis and post-analysis of large phylogenies. Bioinformatics.

[51] Jia B, Raphenya AR, Alcock B, Waglechner N, Guo P, Tsang KK, Lago BA, Dave BM, Pereira S, Sharma AN (2017). CARD 2017: expansion and model-centric curation of the comprehensive antibiotic resistance database. Nucleic Acids Res.

[52] Bengtsson-Palme J, Rosenblad MA, Molin M, Blomberg A (2014). Metagenomics reveals that detoxification systems are underrepresented in marine bacterial communities. BMC Genomics.

[53] Finn RD, Coggill P, Eberhardt RY, Eddy SR, Mistry J, Mitchell AL, Potter SC, Punta M, Qureshi M, Sangrador-Vegas A (2015). The Pfam protein families database: towards a more sustainable future. Nucleic Acids Res.

[54] Johnson LS, Eddy SR, Portugaly E (2010). Hidden Markov model speed heuristic and iterative HMM search procedure. BMC bioinformatics.

[55] Kaminski J, Gibson MK, Franzosa EA, Segata N, Dantas G, Huttenhower C (2015). High-specificity targeted functional profiling in microbial communities with ShortBRED. PLoS Comput Biol.

[56] Leplae R, Lima-Mendez G, Toussaint A (2010). ACLAME: a CLAssification of Mobile genetic Elements, update 2010. Nucleic Acids Res.

[57] Luo F, Zhong J, Yang Y, Scheuermann RH, Zhou J (2006). Application of random matrix theory to biological networks. Phys Lett A.

[58] Qin N, Liang P, Wu C, Wang G, Xu Q, Xiong X, Wang T, Zolfo M, Segata N, Qin H, Knight R, Gilbert JA, Zhu TF: Longitudinal survey of microbiome associated with particulate matter in a megacity. NCBI SRA. https://www.ncbi.nlm.nih.gov/sra?linkname=bioproject_sra_all&from_uid=486429 (2019). 10.1186/s13059-020-01964-x PMC7055069 32127018

