# Supplementary material for: Longitudinal survey of microbiome associated with particulate matter in a megacity
Source: Genome Biol. 2020 Mar 3;21:55. doi: 10.1186/s13059-020-01964-x (PMC7055069; doi:10.1186/s13059-020-01964-x)
Supplement: Supplementary file 2 — Additional file 2: Figure S1. Most abundant microorganism species identified from airborne particulate matters (a) and the meteorological factors associated with PM microbiome (b). a, Box plot of the daily variations of the relative abundance of the top 50 most abundant microorganism species in PM samples. Boxes correspond to the interquartile range between the 25th and 75th percentiles, and the central lines represent the 50th percentile. Ends of the central lines correspond to the lowest and highest values no more than 1.5 times the interquartile range from the box, while circles represent the outliers. Red, PM2.5; Blue, PM10. b, The bar plot shows the explained variation of each factor in the variation of microbial composition [Bray–Curtis (BC) distance]. c. Spearman rank-order correlation plot showing the relationship between pathogen mapped reads (%) and PM concentration. Figure S2. Temporal distribution of the daily relative abundance of 96 human pathogens and PM concentration variations during the sampling time. Figure S3. Strain-level phylogenetic trees of Escherichia coli. Black, reference strains; red, MetaSUB samples; green, PM samples. Figure S4. Strain-level phylogenetic trees of Propionibacterium acnes (a), Acinetobacter lwoffi (b) and Pantoea ananatis (c). Black, reference strains; red, MetaSUB samples; green, PM samples. Figure S5. Strain-level phylogenetic trees of Kocuria sp. UCD OTCP (a), Acinetobacter johnsonii (b) and Pantoea dispersa (c). Black, reference strains; red, MetaSUB samples; green, PM samples. Figure S6. Strain-level phylogenetic trees of Rhodococcus sp. R04. Black, reference strains; red, MetaSUB samples; green, PM samples. Figure S7. Comparison of genesets from PM, ocean and gut microbiota (a, b, c) and the network topological variables of PM microbiota (d, e). a, Venn diagram indicating a low overlap of PM, human gut and ocean gene catalog. b, Venn diagram of core OGs suggesting a large overlap of functions among PM, human gut and [file 13059_2020_1964_MOESM2_ESM.docx]

Additional file 2: Supplementary figures for:

**Longitudinal survey of particulate matter associated microbiome in a megacity**

**Supplementary Figures**

**
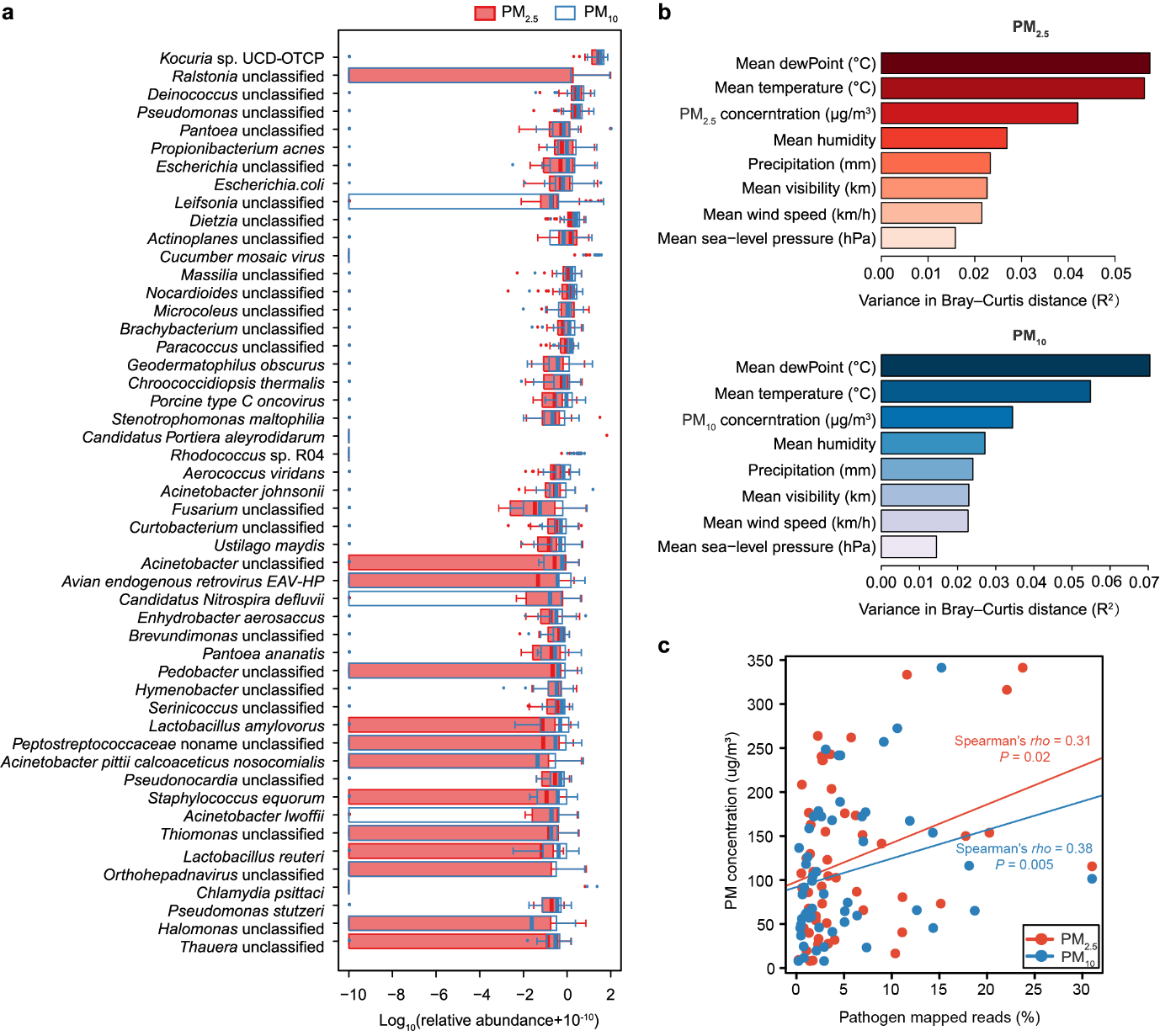
**

**Figure S1. Most abundant microorganism species identified from airborne particulate matters (a) and the meteorological factors associated with PM microbiome (b). a,** Box plot of the daily variations of the relative abundance of the top 50 most abundant microorganism species in PM samples. Boxes correspond to the interquartile range between the 25th and 75th percentiles, and the central lines represent the 50th percentile. Ends of the central lines correspond to the lowest and highest values no more than 1.5 times the interquartile range from the box, while circles represent the outliers. Red, PM_2.5_; Blue, PM_10_. **b,** The bar plot shows the explained variation of each factor in the variation of microbial composition [Bray–Curtis (BC) distance]. **c.** Spearman rank-order correlation plot showing the relationship between pathogen mapped reads (%) and PM concentration.

**
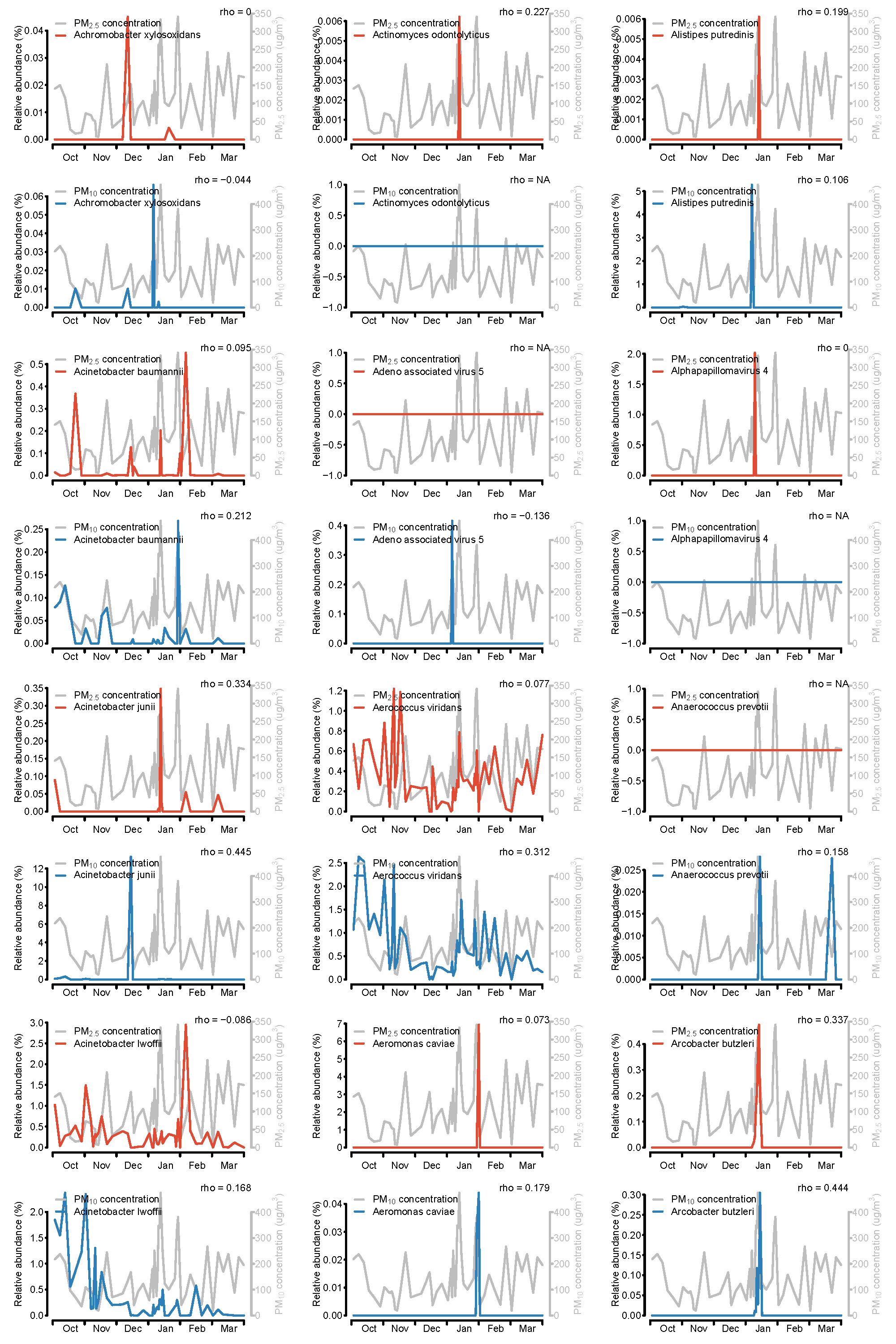

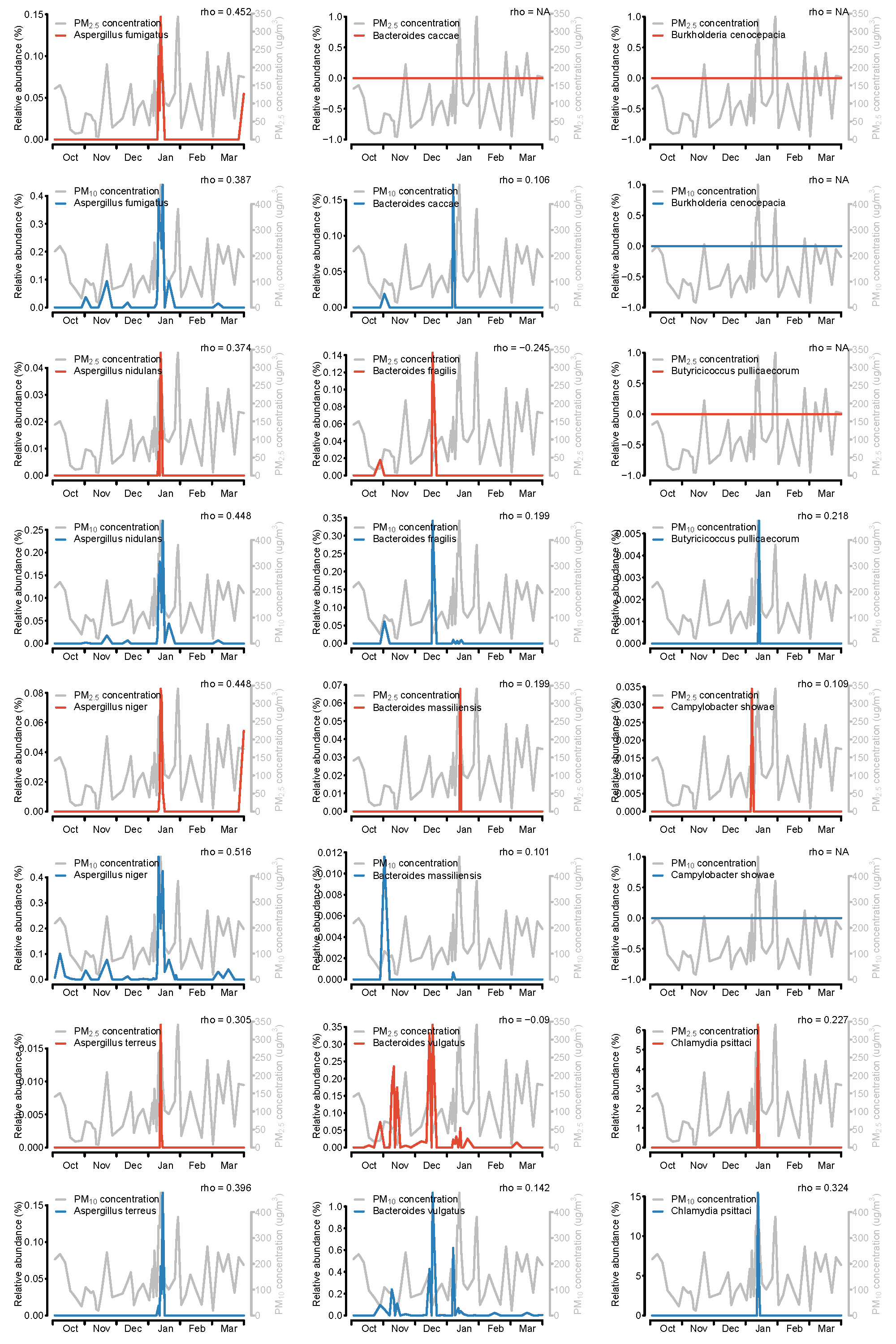

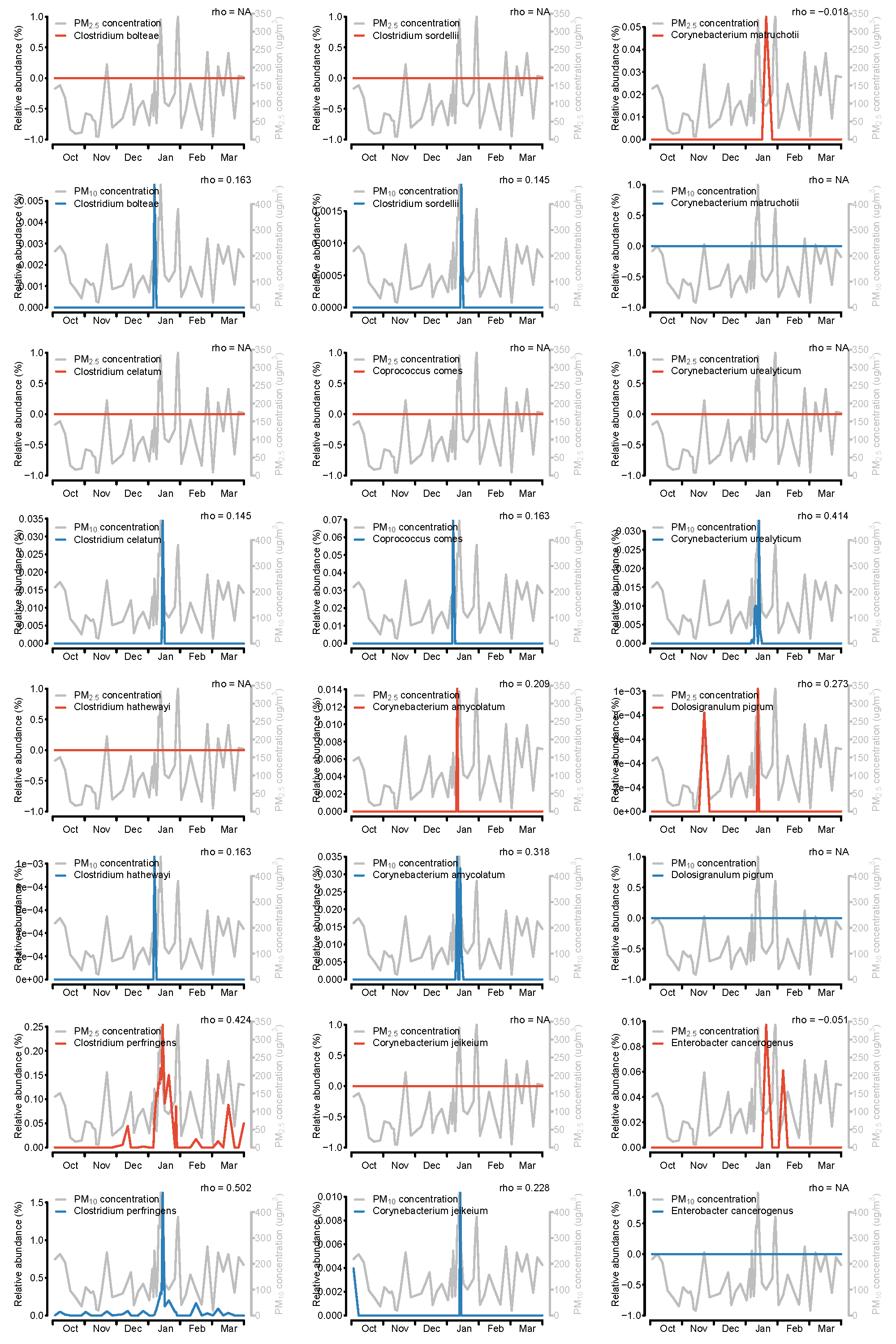

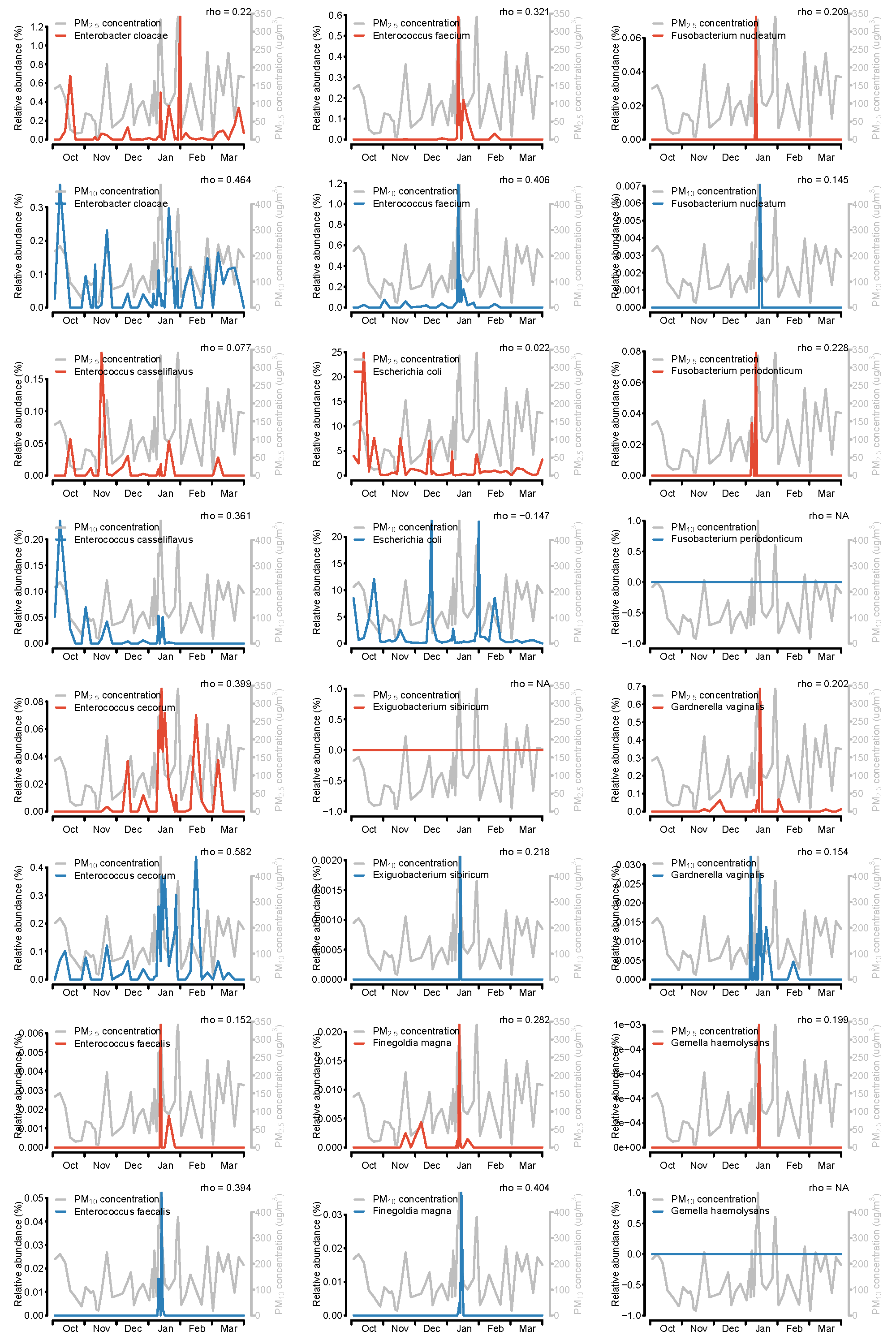

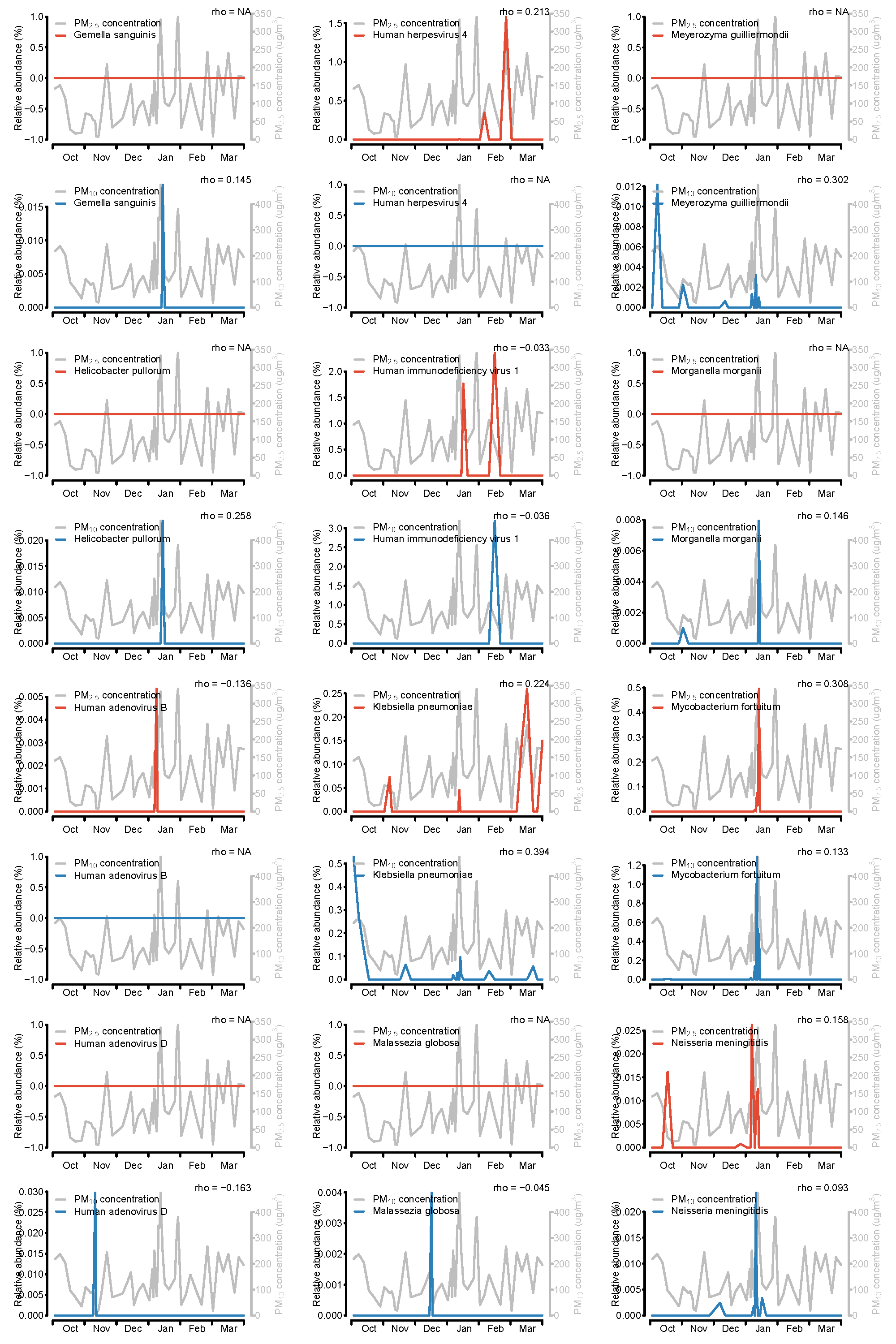

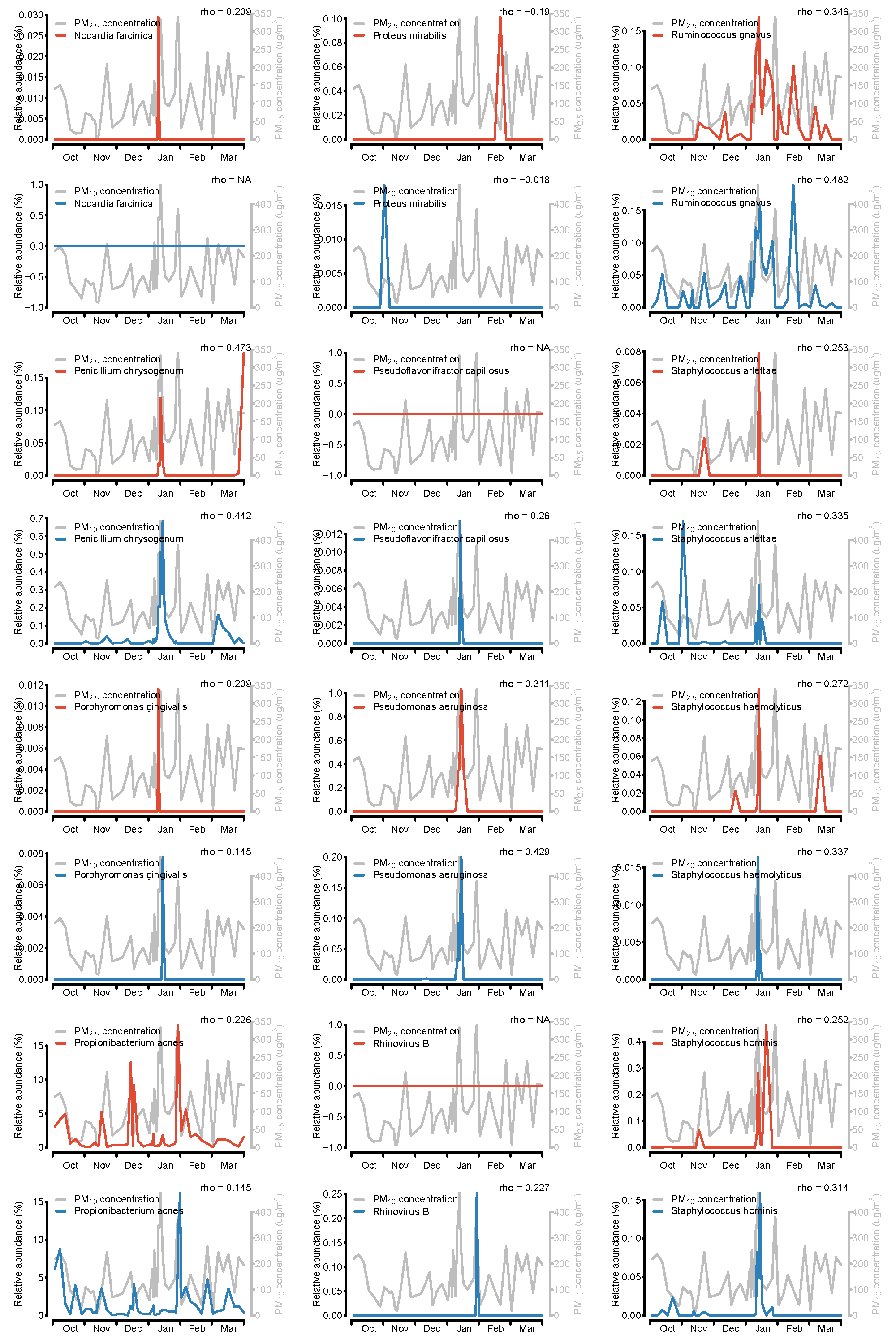

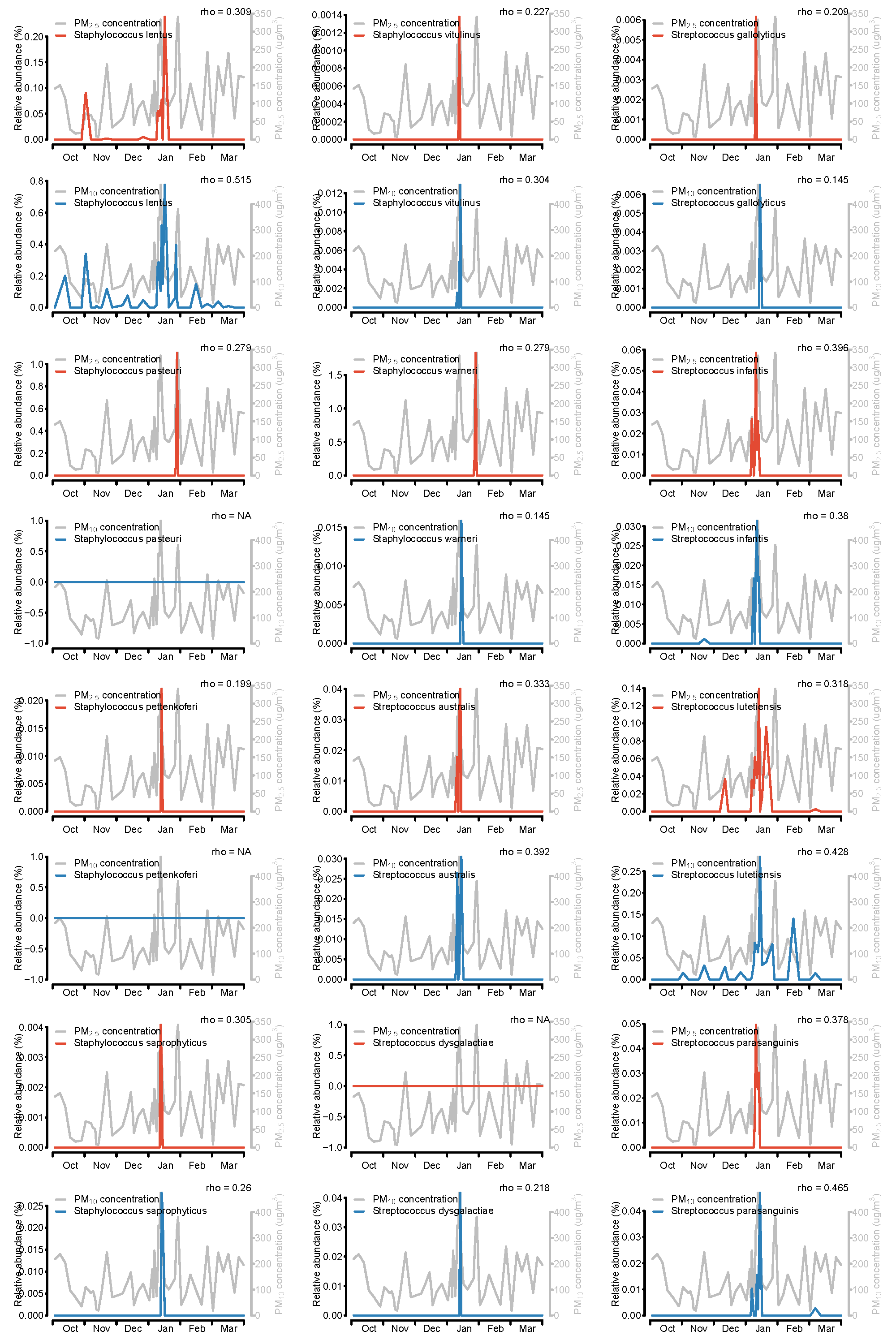

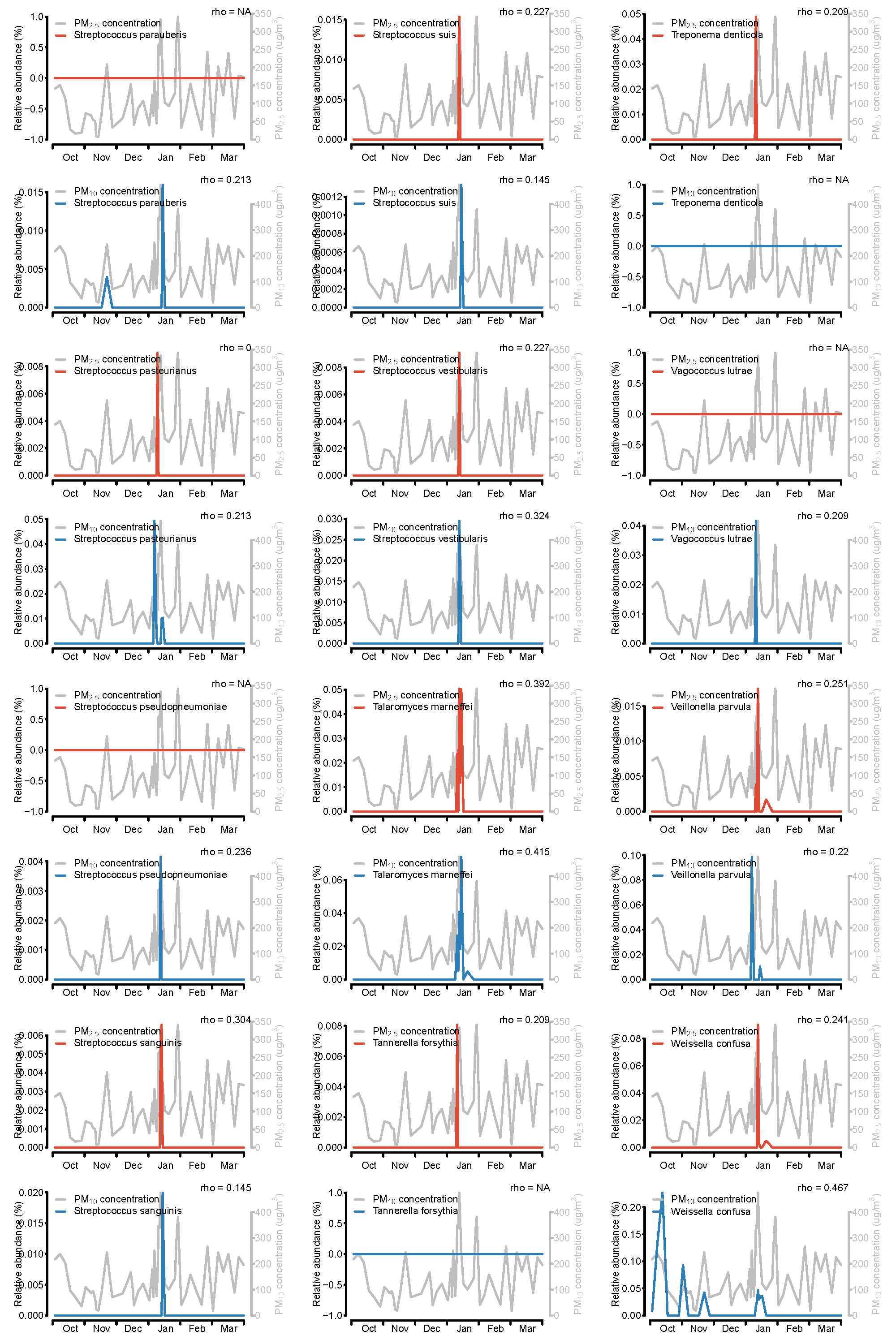
**

**Figure S2. Temporal distribution of the daily relative abundance of 96 human pathogens and PM concentration variations during the sampling time.**

**
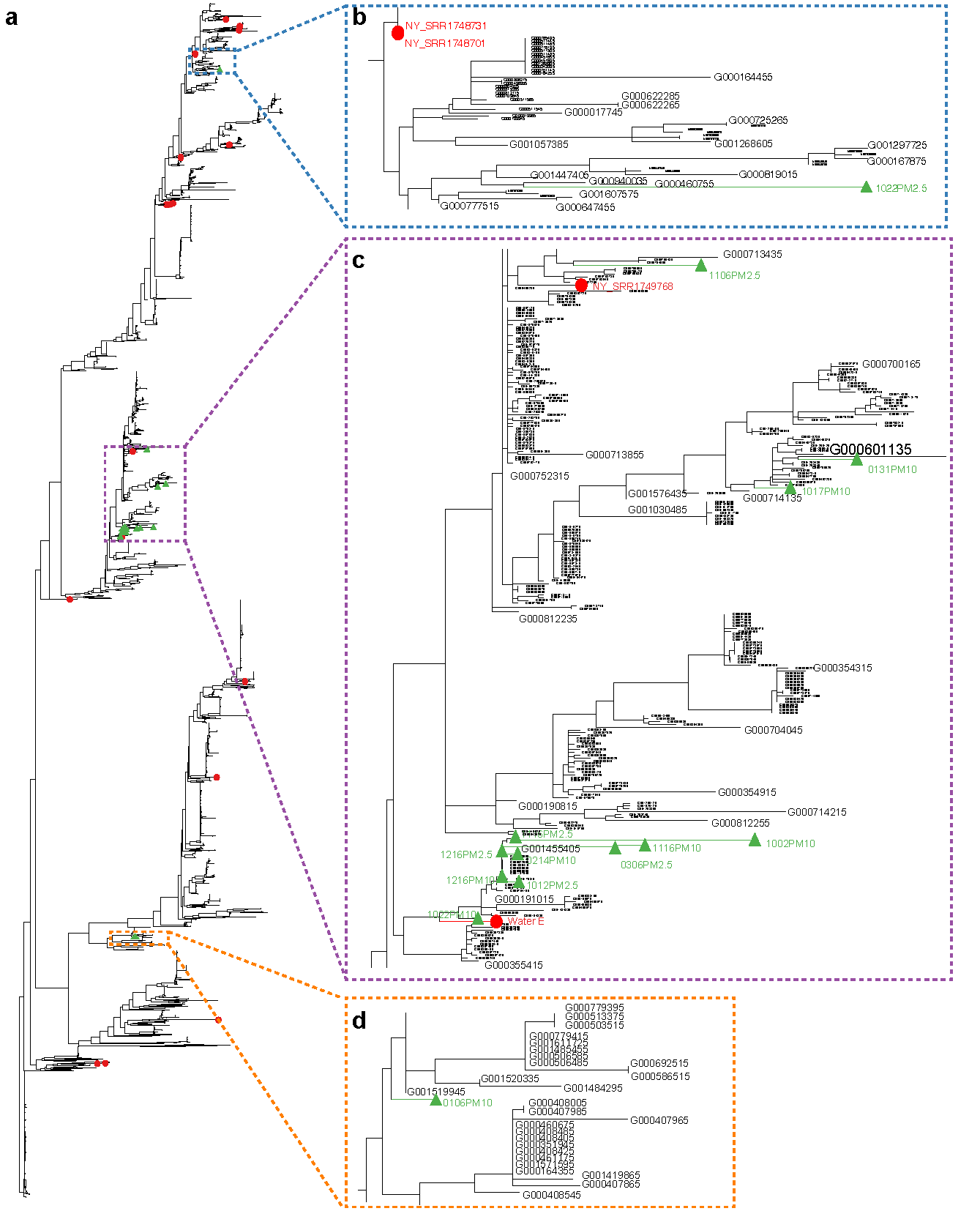
**

**Figure S3.** **Strain-level phylogenetic trees of *Escherichia coli*.** Black, reference strains; red, MetaSUB samples; green, PM samples.

**
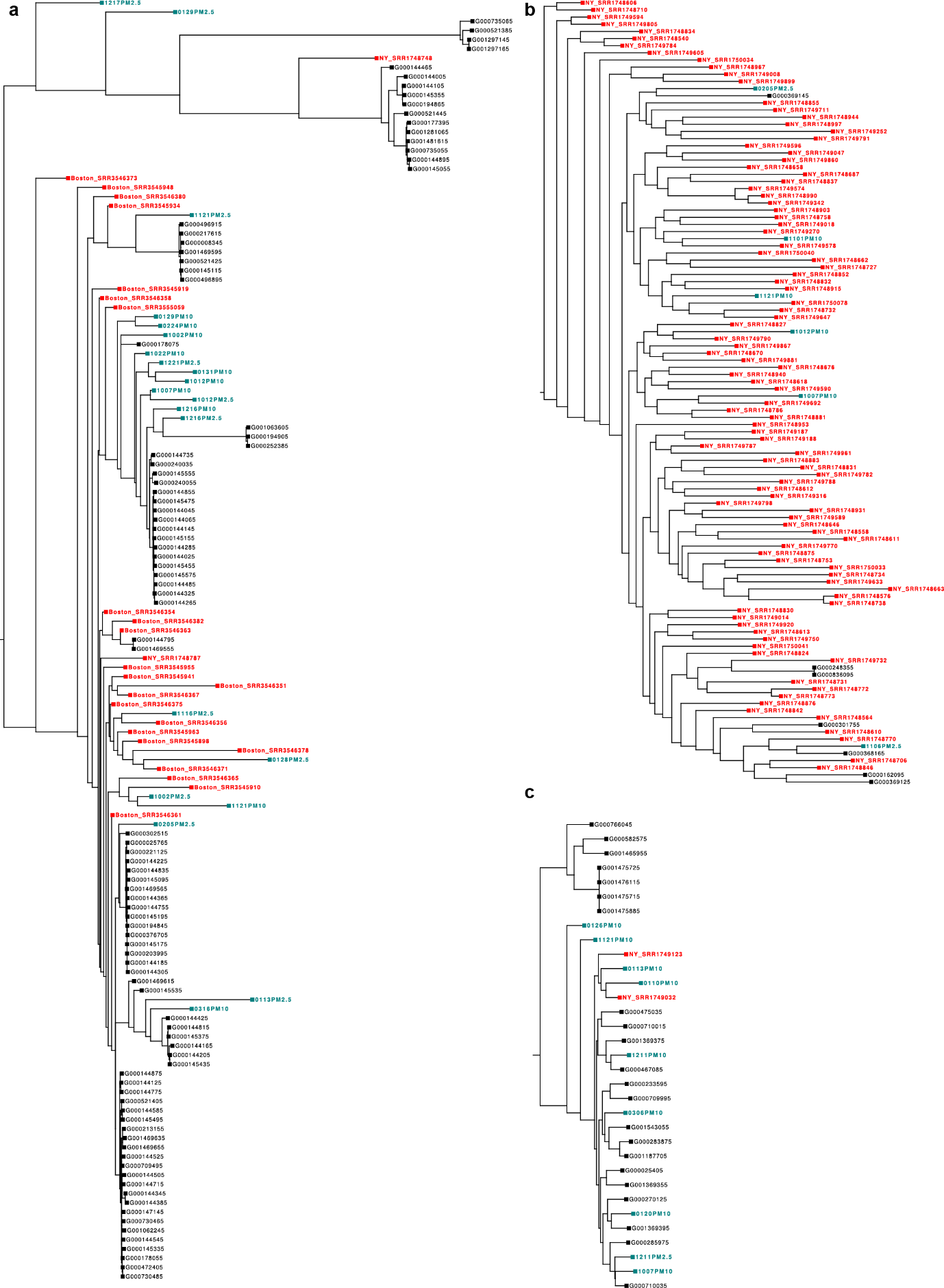
**

**Figure S4.** **Strain-level phylogenetic trees of *Propionibacterium acnes* (a), *Acinetobacter lwoffi* (b) *and Pantoea ananatis* (c)*.*** Black, reference strains; red, MetaSUB samples; green, PM samples.


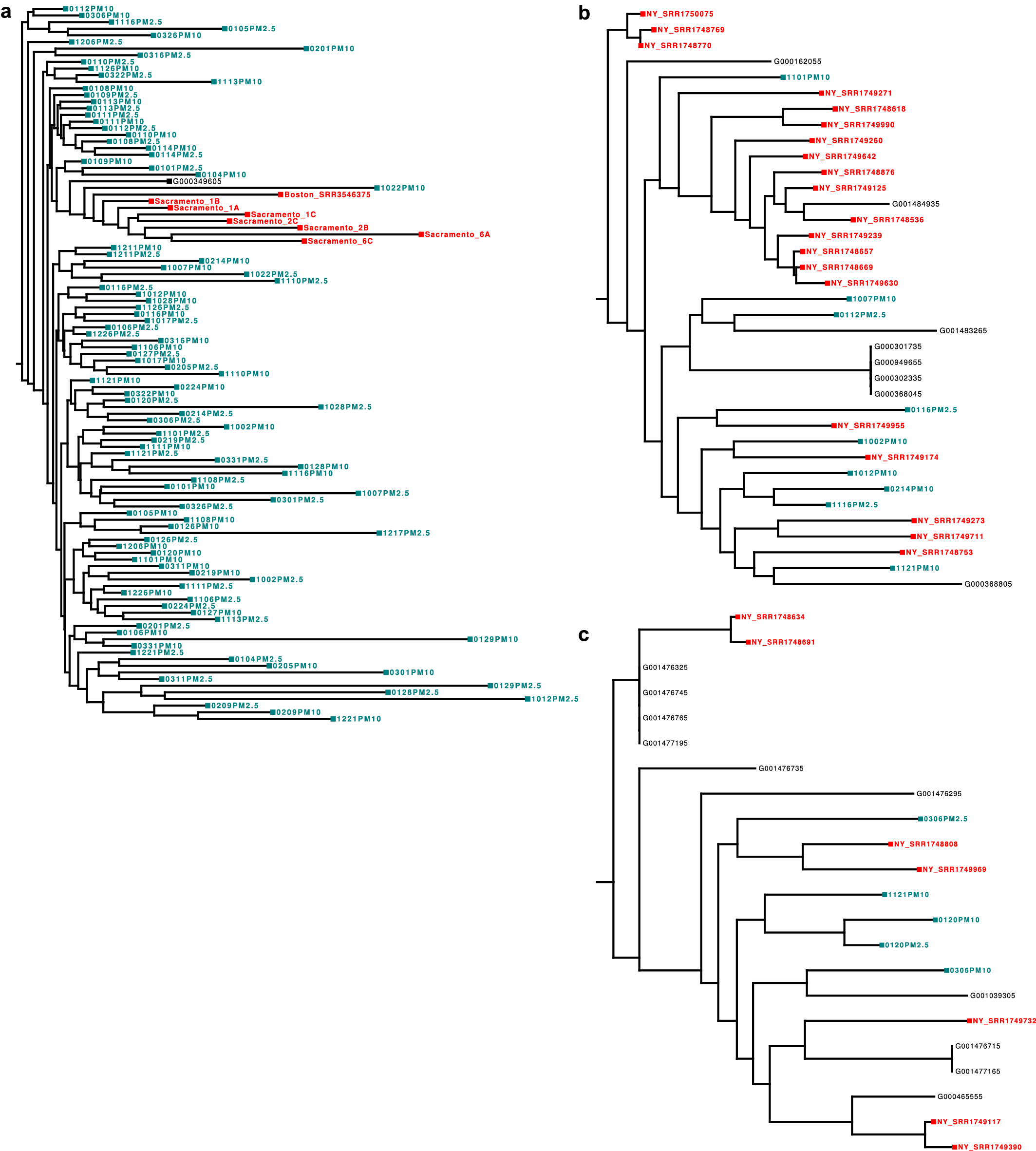


**Figure S5.** **Strain-level phylogenetic trees of *Kocuria* sp. UCD OTCP (a), *Acinetobacter johnsonii* (b) and *Pantoea dispersa* (c).** Black, reference strains; red, MetaSUB samples; green, PM samples.


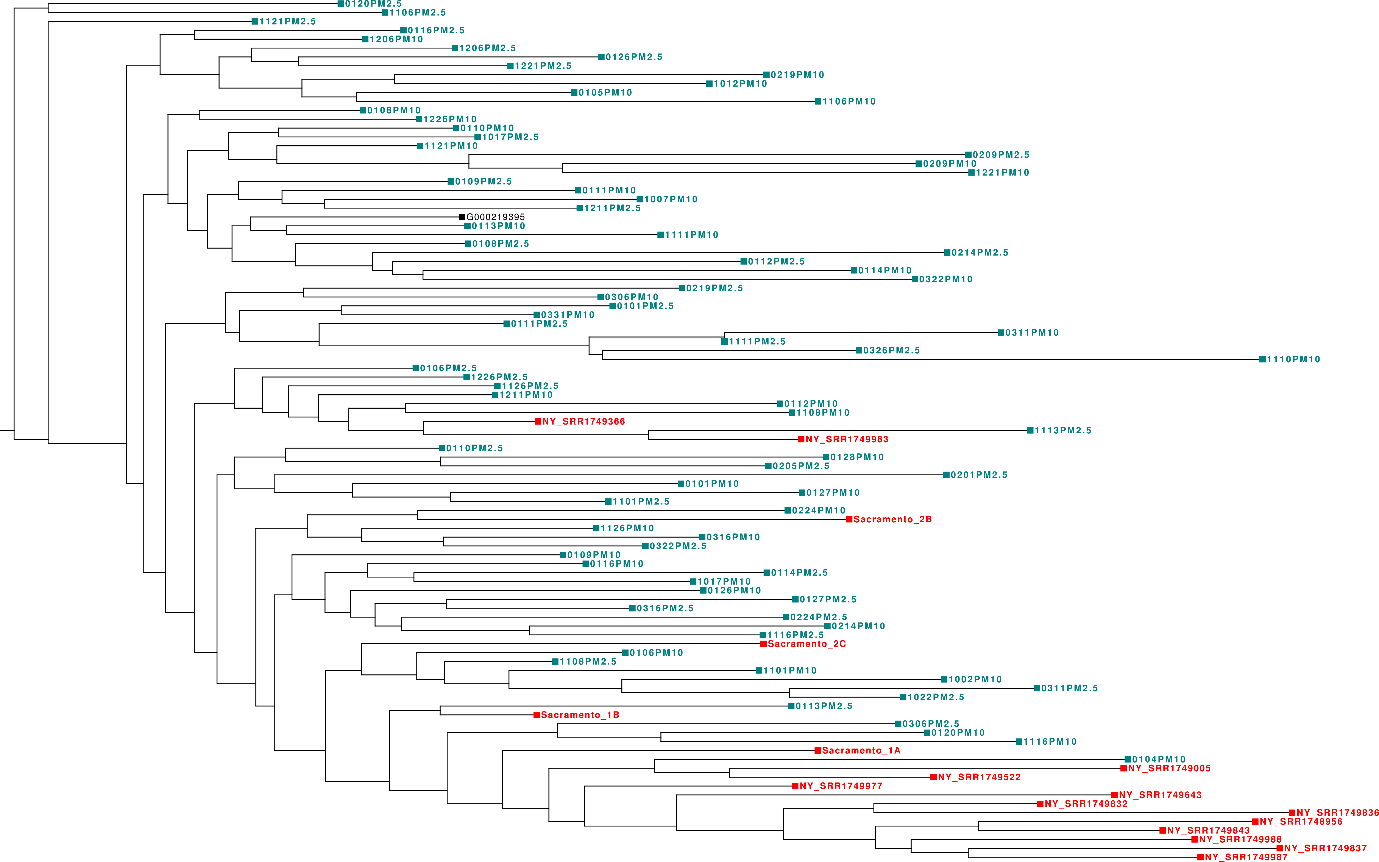


**Figure S6.** **Strain-level phylogenetic trees of *Rhodococcus* sp. R04.** Black, reference strains; red, MetaSUB samples; green, PM samples.

**
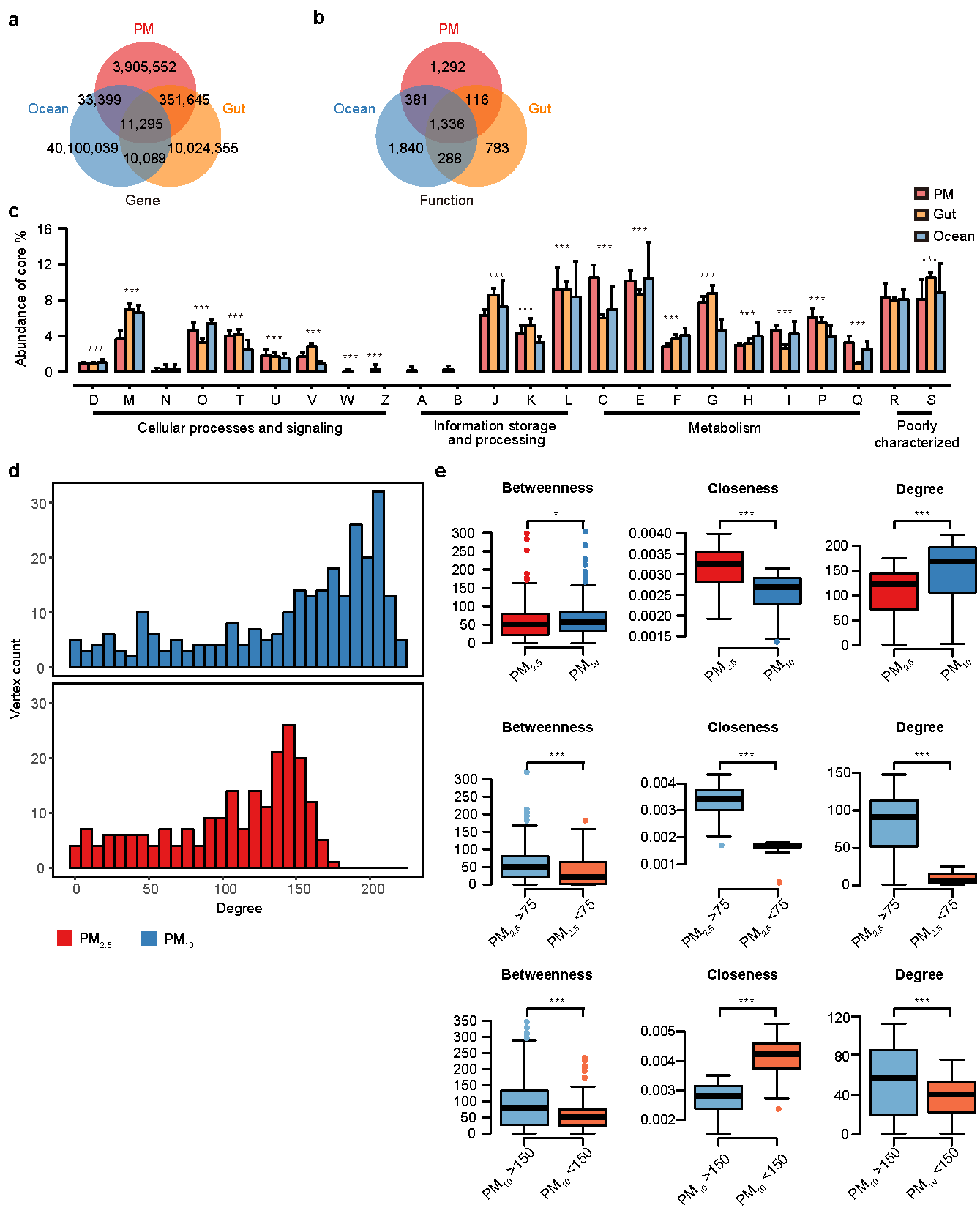
**

**Figure S7.** **Comparison of genesets from PM, ocean and gut microbiota (a, b, c) and the network topological variables of PM microbiota (d, e). a,** Venn diagram indicating a low overlap of PM, human gut and ocean gene catalog. **b,** Venn diagram of core OGs suggesting a large overlap of functions among PM, human gut and ocean microbiota. **c**, Bar chart showing the comparison of gene abundance summarized into OG functional categories. A, RNA processing and modification; B, Chromatin structure and dynamics; C, Energy production and conversion; D, Cell cycle control, cell division, chromosome partitioning; E, Amino acid transport and metabolism; F, Nucleotide transport and metabolism; G, Carbohydrate transport and metabolism; H, Coenzyme transport and metabolism; I, Lipid transport and metabolism; J, Translation, ribosomal structure and biogenesis; K, Transcription; L, Replication, recombination and repair; M, Cell wall/membrane/envelope biogenesis; N, Cell motility; O, Posttranslational modification, protein turnover, chaperones; P, Inorganic ion transport and metabolism; Q, Secondary metabolites biosynthesis, transport and catabolism; R, General function prediction only; S, Function unknown; T, Signal transduction mechanisms; U, Intracellular trafficking, secretion, and vesicular transport; V, Defense mechanisms; W, Extracellular structures; Z, Cytoskeleton. **d,** The distribution of degree of nodes in the networks. **e,** Network topological variables comparison between PM_2.5_ (red) and PM_10_ (blue) samples (up), PM_2.5_ <75 μg/m^3^ and PM_2.5_ >75 μg/m^3^ (middle), as well as PM_10_ <150 μg/m^3^ and PM_10_ >150 μg/m^3^ (bottom). Asterisks denote Kruskal-Wallis test results, p-values were adjusted using Benjamini and Hochberg false discovery rate (FDR) (*, adjusted *P* <0.05, **, adjusted *P* <0.01, ***, adjusted *P* <0.001).


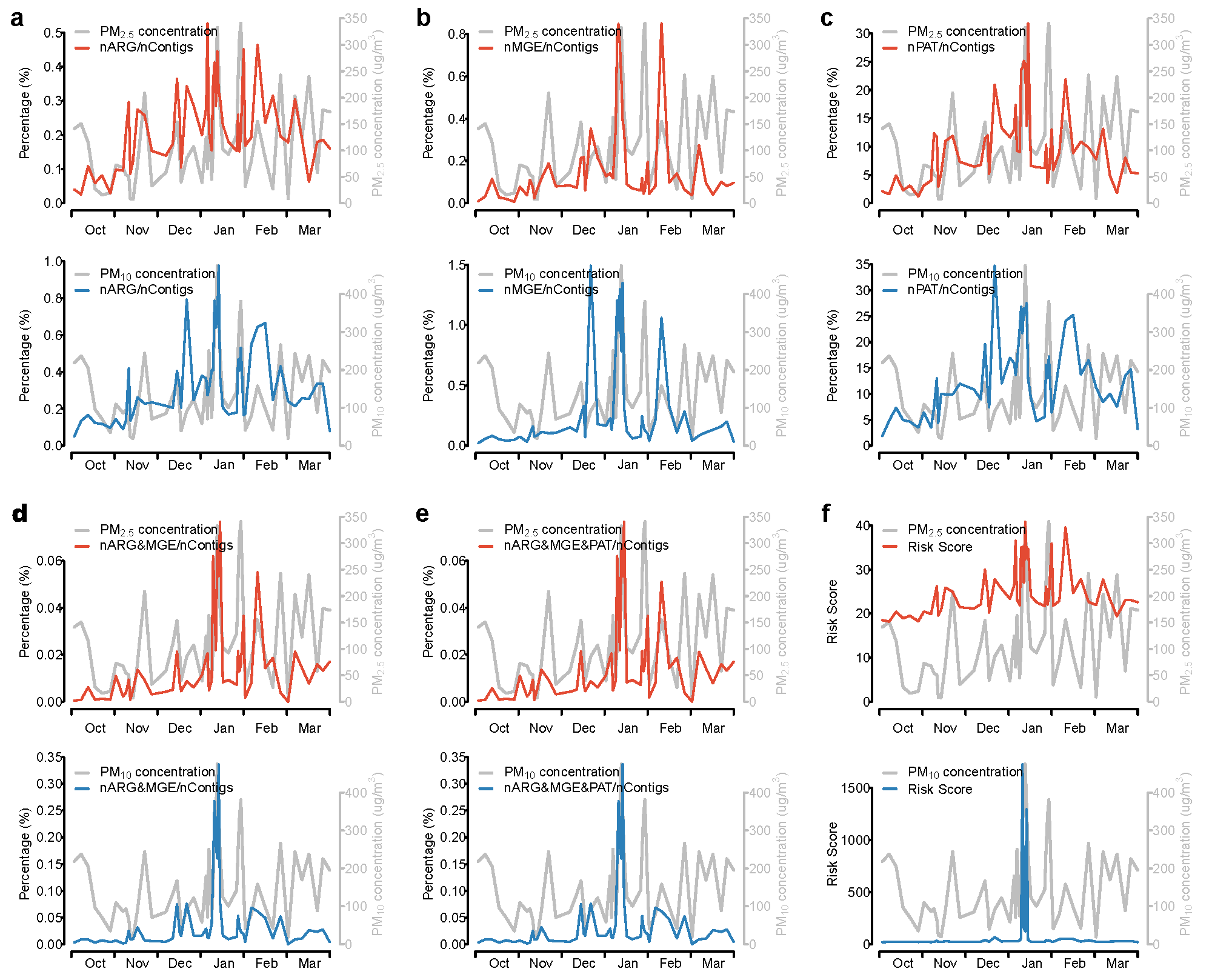


**Figure S8. Temporal distribution of daily resistance risk and PM concentration variations during the sampling time.** (a-e) The percentage of contigs with ARG (a), MGE (b), pathogen (c), ARG&MGE (d) and ARG&MGE&pathogen (e) in all contigs. (f) The resistance risk score.

**
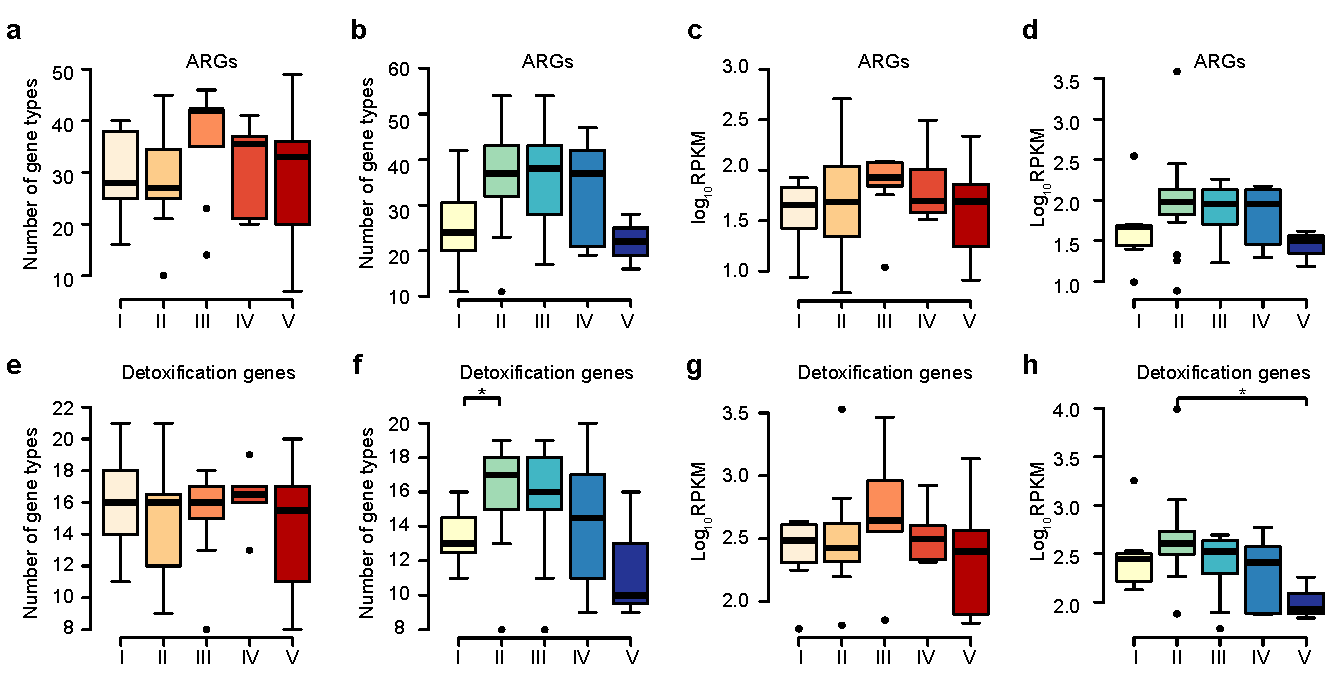
**

**Figure S9. Comparative analyses of antibiotic resistance and detoxification gene for 5 different classes of PM_2.5_ and PM_10_ samples.** **a, b, c, d,** show the numbers of antibiotic resistance gene types (a, b) and RPKM values of the total antibiotic resistance gene types (c, d) in PM_2.5_ (red) and PM_10_ (blue) samples, respectively. **e, f, g, h**, show the numbers of detoxification gene types (e, f) and RPKM values of the total detoxification gene types (g, h) in PM_2.5_ (red) and PM_10_ (blue) samples, respectively. Asterisks denote Kruskal-Wallis test results, p-values were adjusted using Benjamini and Hochberg false discovery rate (FDR) (*, adjusted *P* <0.05).


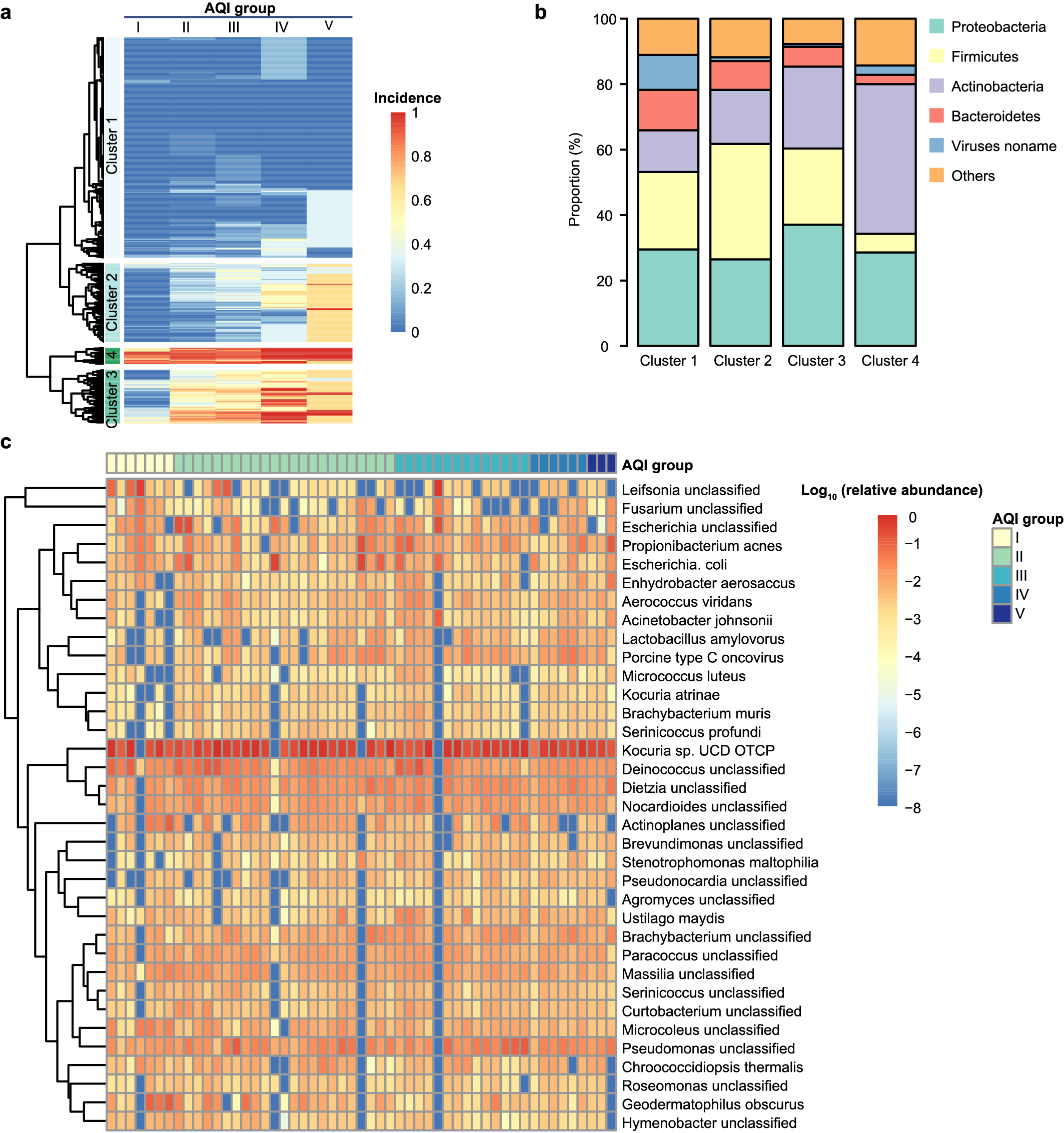


**Figure S10. The species clusters based on their incidence patterns in the five PM concentration levels of PM_10_ samples. a,** Hierarchical Ward-linkage clustering of species based on their incidence patterns in the five PM concentration levels of PM_10_ samples. Colouring represents the incidence (per sample detection rate). **b**, Proportion of species affiliating to each of the 5 phyla in the four species clusters of PM_10_ samples. **c**, The heat map of relative abundance of species in cluster 4. Colouring represents the relative abundance of species.


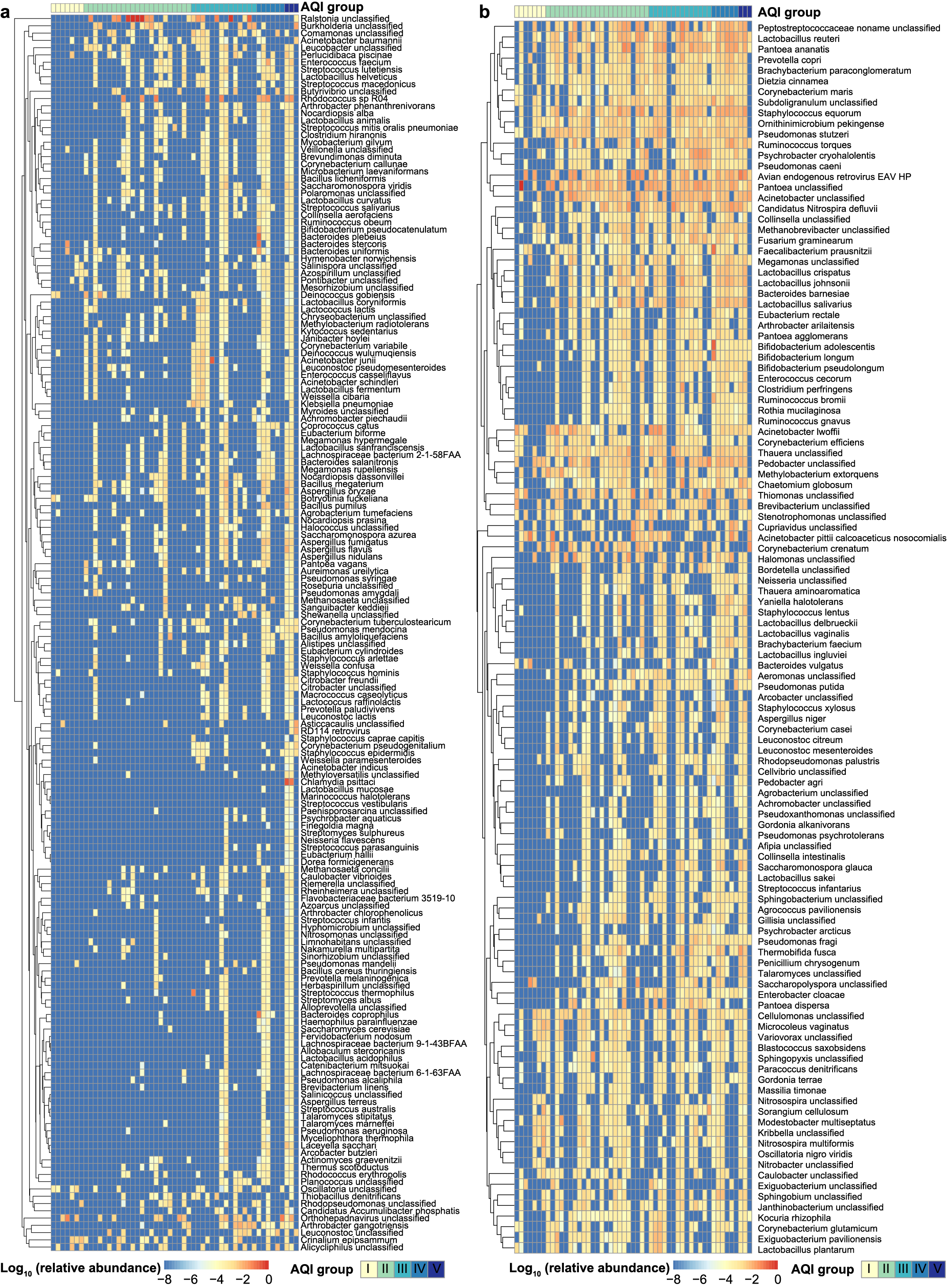


**Figure S11. The heat map of relative abundance of species in cluster 2 (a) and cluster 3 (b).**

**
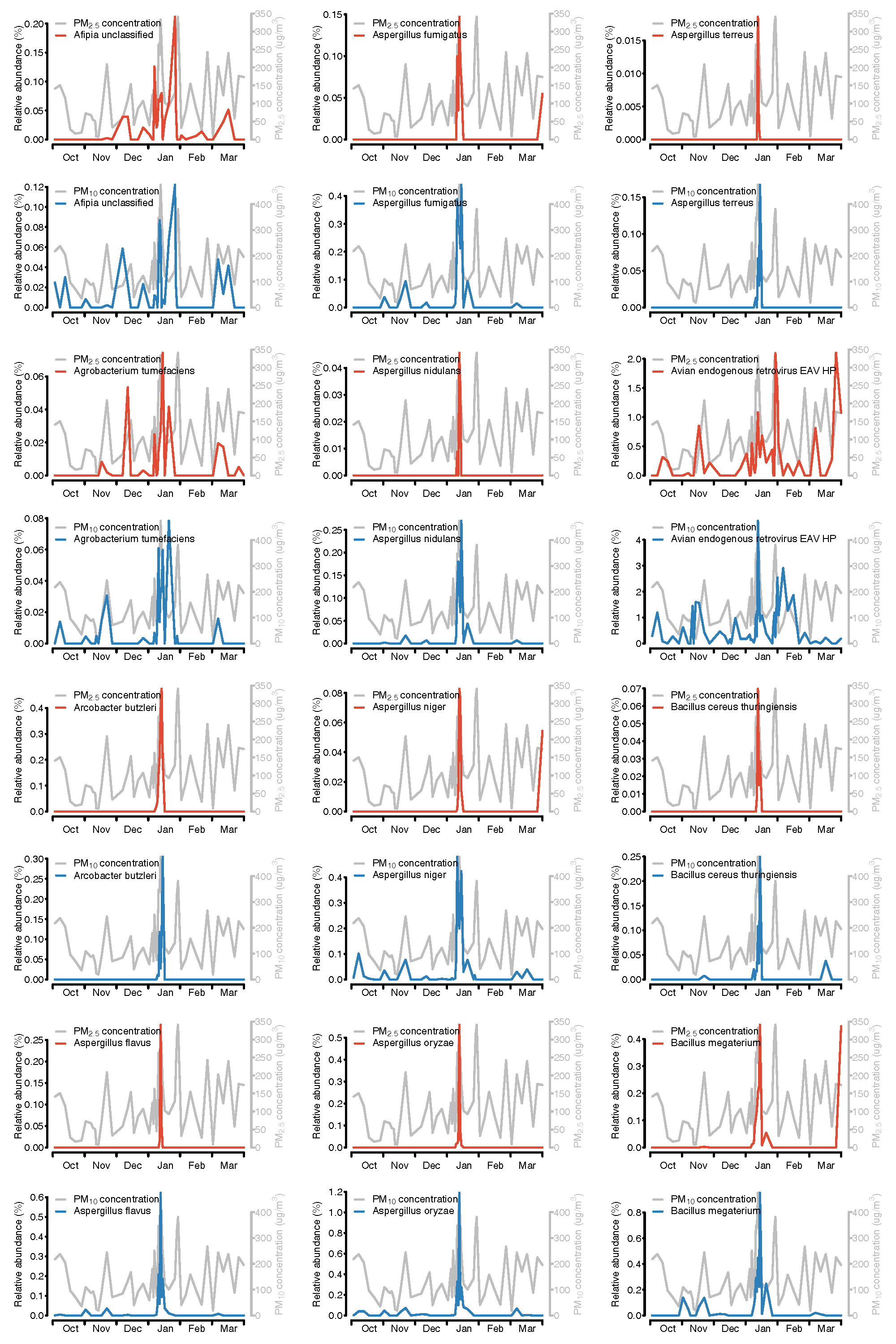
**

**
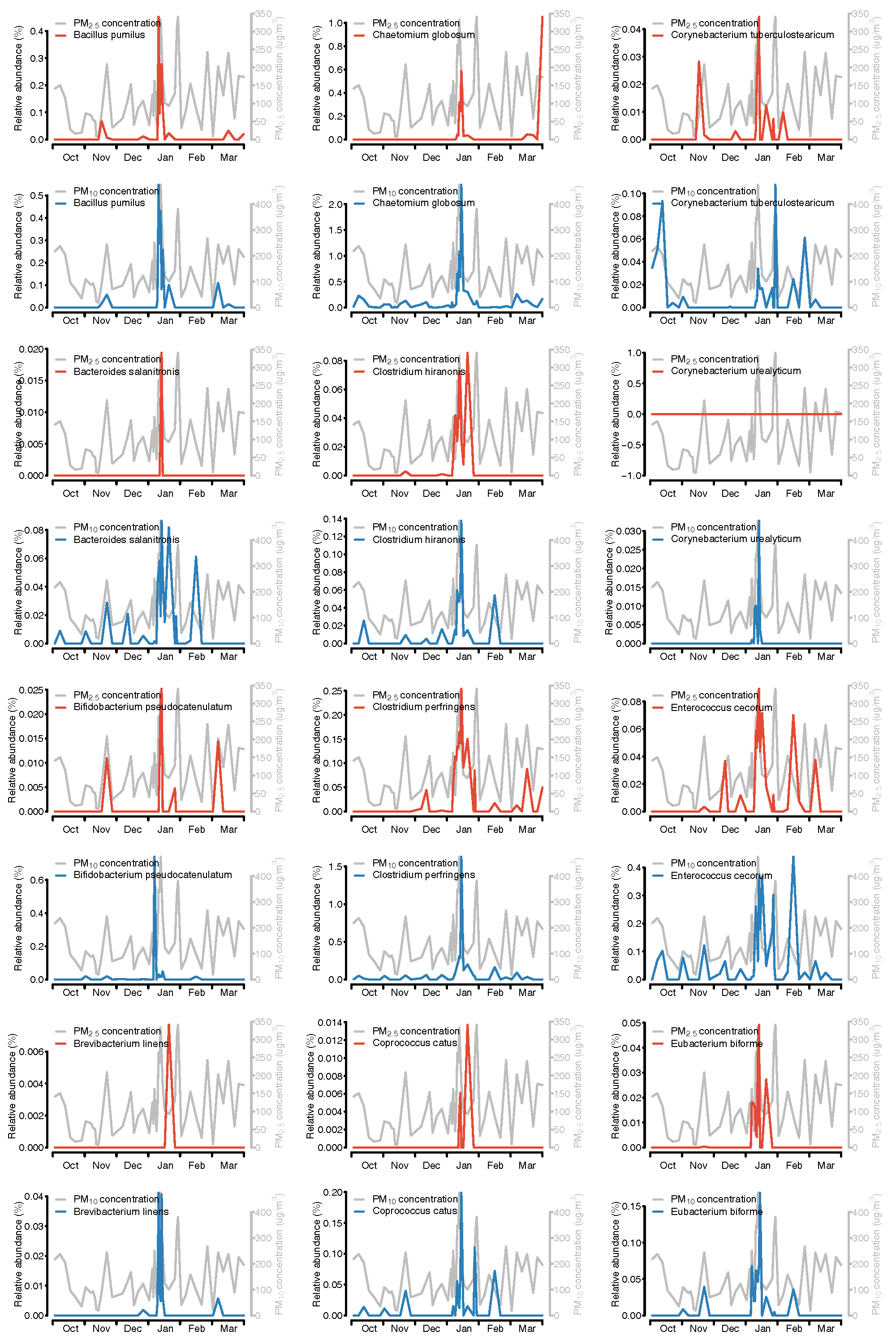

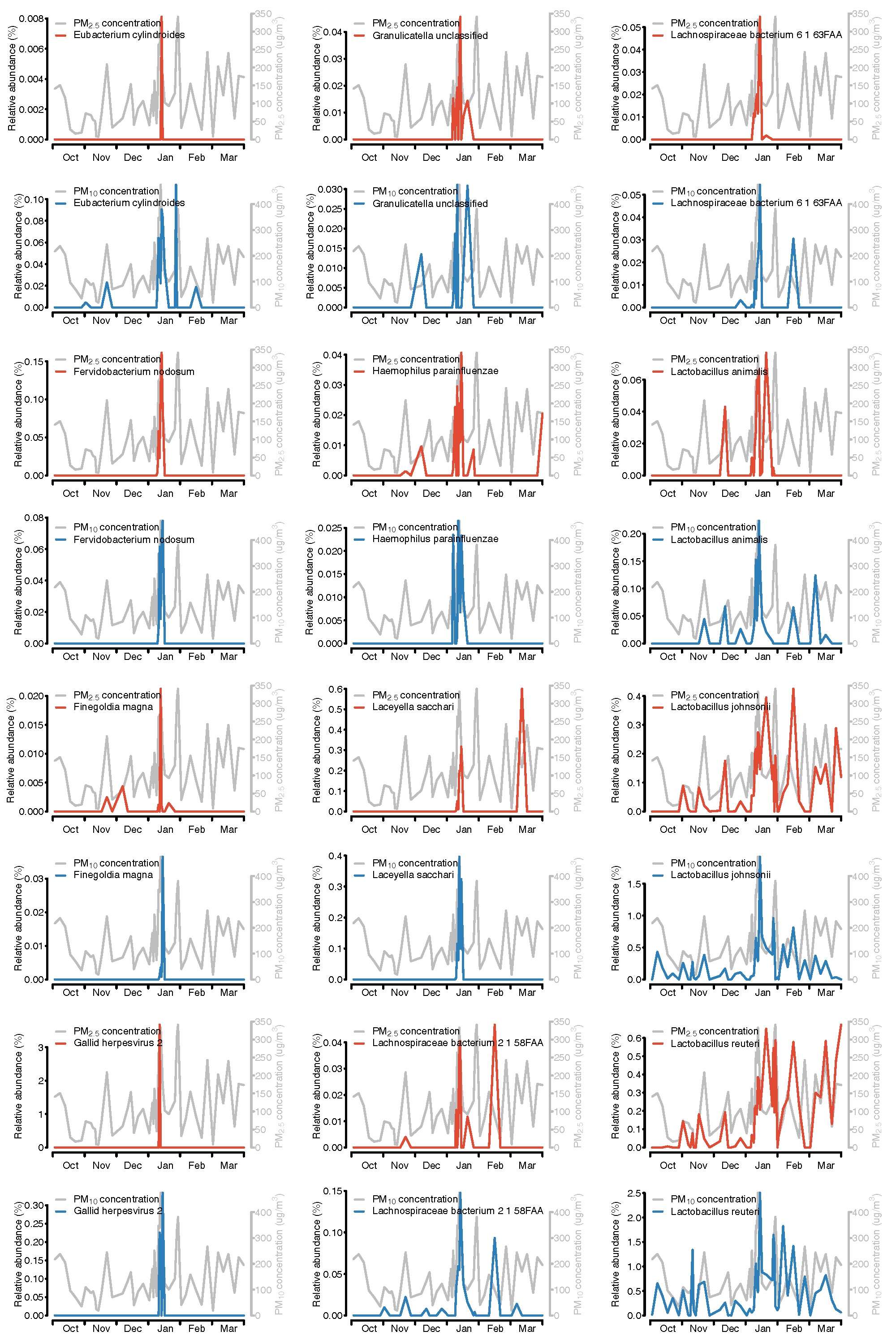

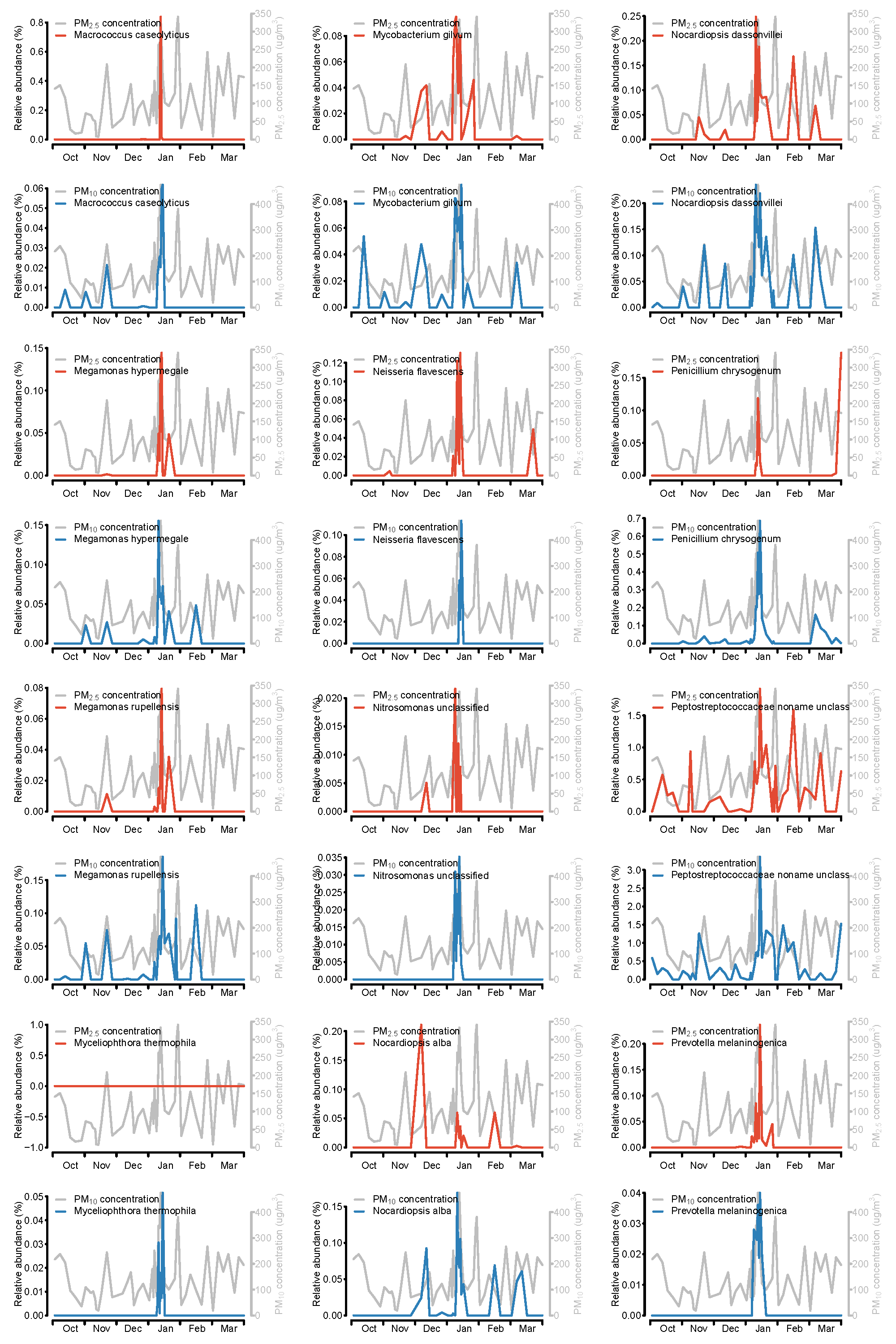

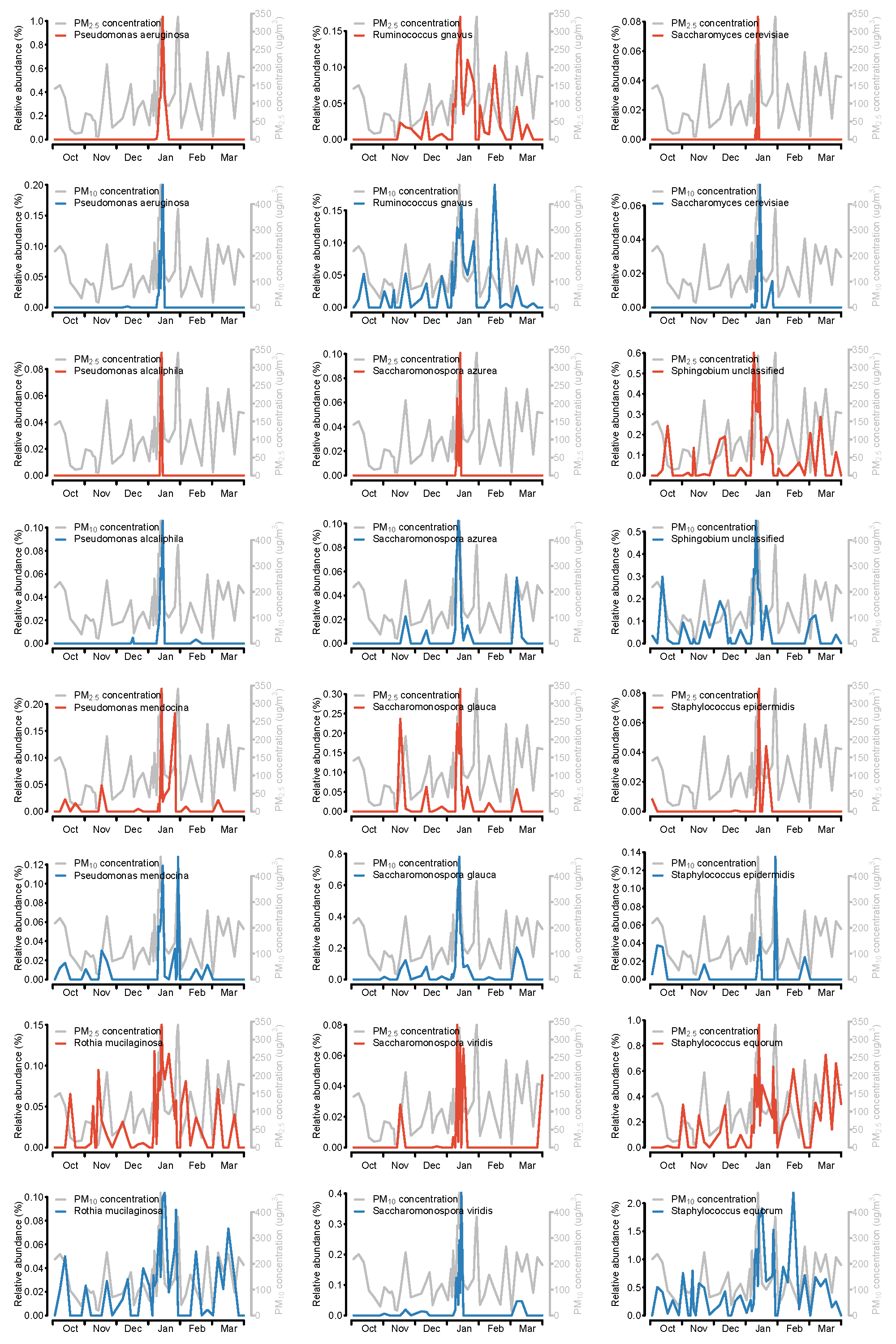

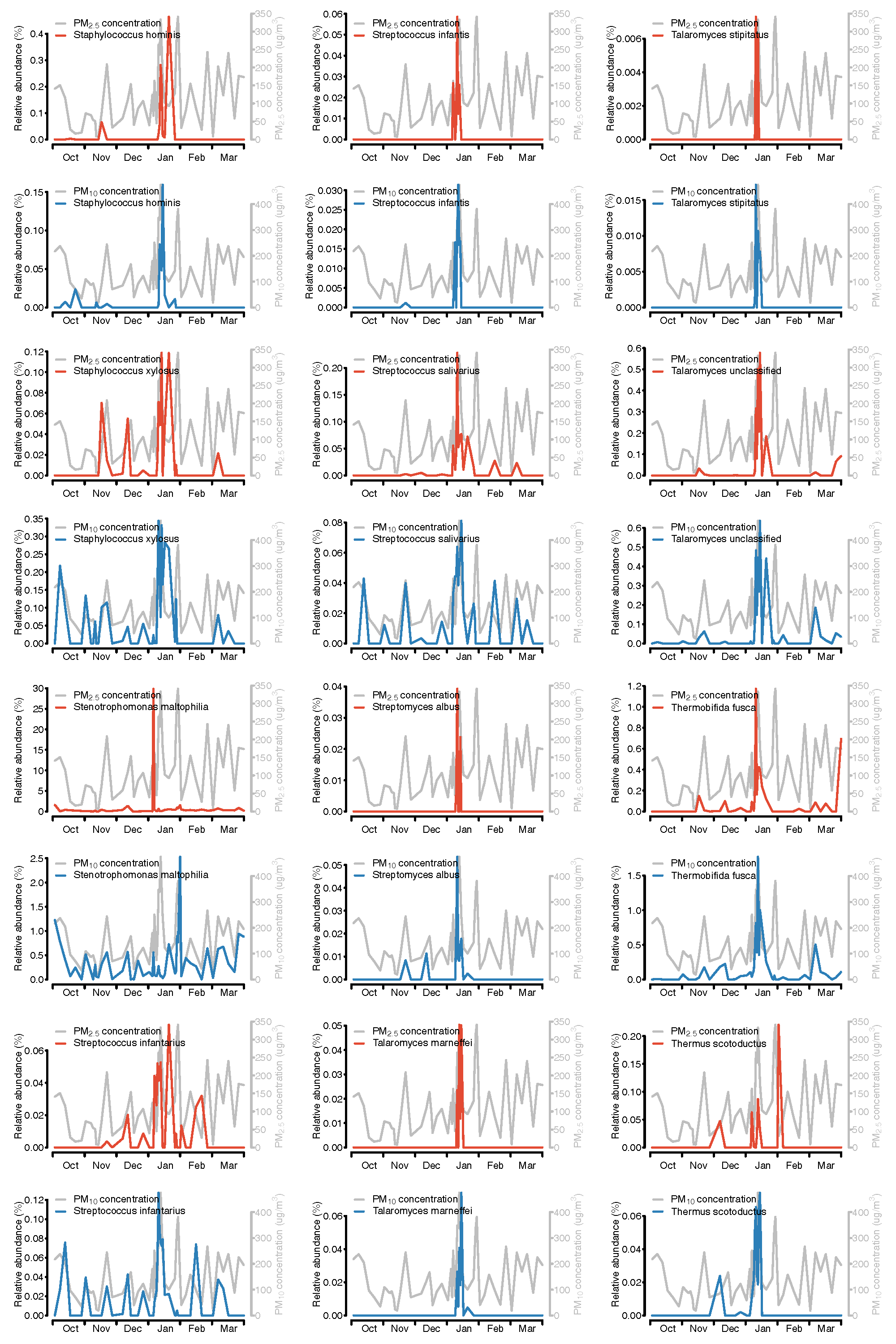
**

**Figure S12. Temporal distribution of the daily relative abundance of 72 microbes and PM concentration variations during the sampling time.**
