# Supplementary material for: Longitudinal survey of microbiome associated with particulate matter in a megacity
Source: Genome Biol. 2020 Mar 3;21:55. doi: 10.1186/s13059-020-01964-x (PMC7055069; doi:10.1186/s13059-020-01964-x)
Supplement: Supplementary file 3 — Additional file 3. Review history. [file 13059_2020_1964_MOESM3_ESM.docx]

Review History

**First round of review**

**Reviewer 1**

**Are you able to assess all statistics in the manuscript, including the appropriateness of statistical tests used?** Yes, and I have assessed the statistics in my report.

**Comments to author:**

In the paper "Longitudinal survey of particulate matter associated microbiome in a megacity", Qin and co-authors investigate the microbiome in air particles collected in Beijing over six months. Shotgun metagenomics is used to characterize and functionally analyze the microbial communities. The authors show that communities show similarities between particle size but that there are large temporal variations. They also show the presence of antibiotic resistance genes and a correlation between air quality index and specific species.

The study is overall well designed where a large number of samples have been collected over half a year. The main weakness in the experimental design is that one single site has used for the sampling, which likely reduces the generality of the results. The data analysis is also sound, although there are parts that lack the all the necessary details for repeatability, in particularly those related to the statistics (see detailed comments below). These details need to be provided before the paper can be published.

The introduction and results sections of the papers are well-written and clear. The discussion part is however extremely short and contains only a single paragraph. The conclusion is also very short and contains only a single sentence. Most of the findings described in the paper are therefore not discussed and put into a proper context, both regards to impact and existing research in the field. The authors need to significantly extend the discussion and conclusion before the paper can considered for publication.

Detailed comments
Throughput the paper the authors adjust their p-values to avoid issues with multiple testing. There is however no details on how this was done. What method was used and how there the p-values adjusted? Provide information if adjustment was done per analysis (how many analyses in total) or global. Also, no adjustment seems to have been done for the large number of tests in Supplementary Figure S7 and S8.
Line 95-98: How were the correlations between taxonomic composition and meterological factors computed? The R2 values presented in Table S5 suggests a parametric model. However, the values are all extremely low indicating overall low correlations. Please provide the full details. Also, why were these p-values not adjusted for multiple testing?
Line 116-119: How were the tests comparing the gene abundances between the three catalogues performed? Pair-wise comparisons? Please provide more information.
Line 123: "Genes that potentially confer resistance to 9 different antibiotics were identified, which can be further divided into 22 different classes". What do the authors mean with "9 different antibiotics"? Types of antibiotics? And I assume that it was the genes that could be divided into 22 classes, not the antibiotics? Please rephrase.
Line 129: "MFS categories" -> "MFS transporter categories"
Line 134: The low proportion of MGEs found next to ARGs shows that either 1) the authors failed to assemble the MGEs properly or 2) the authors ARG database contains a lot of non-ARG genes. Please elaborate on why this number is so low.
Line 152-153: This sentence is misleading. The only significance is between group I and II for PM10. There is no evidence of a general pattern.
Line 191-193: ARGs are commonly present in all forms of bacterial communities. This study have not show that Beijing smog contains any ARGs of particular concern so this conclusion should be toned down.
Line 304-305: What does "the ARG database created using ResFAMs" mean? Also, how exactly were Uniprot used? It is well-known that public databases, such as Uniprot, contains a lot of incorrectly annotated resistance genes. The authors should instead use a well-established ARG database, such as ResFinder or CARD?
Supplementary material: Figure S3 lacks resolution which makes it almost impossible to interpret.

**Reviewer 2**

**Are you able to assess all statistics in the manuscript, including the appropriateness of statistical tests used?** Yes, and I have assessed the statistics in my report.

**Comments to author:**

The manuscript of Qin at al is investigating the composition and functional potential of microbes residing on dust particles in an urban environment over time. The experimental design is sound and the analysis follows standard practices.  It is difficult to extrapolate how the temporal variation identified in Beijing would appear in different cities across the globe. Are those profiles signature-like for different cities or unified by an underlying external factor? A future study based on the same rigorous methodological approach would be able to shed light into such questions.
I only have minor remarks concerning this work.
1)     It would be interesting to know the number of living microbes (non-viral) in the  community. Is there literature known on dust particles plate colonies count or live-dead counts?
2)     What percent of the reads was not mapped by MetaPhlAn2?
3)     Negative controls are not mentioned. It would be interesting to see what the profile of a filter remaining in the sealed bag for a day and then followed the same treatment would look like.
4)     The sequence data for the project are not retrievable yet. Only the project is registered.
5)     Line102 conincided should be coincided.
6)     Line 359 beteween should be between
7)     Line 368 beteween should be between

**Authors Response**

**Point-by-point responses to the reviewers’ comments:**

Reviewer #1: In the paper "Longitudinal survey of particulate matter associated microbiome in a megacity", Qin and co-authors investigate the microbiome in air particles collected in Beijing over six months. Shotgun metagenomics is used to characterize and functionally analyze the microbial communities. The authors show that communities show similarities between particle size but that there are large temporal variations. They also show the presence of antibiotic resistance genes and a correlation between air quality index and specific species.

The study is overall well designed where a large number of samples have been collected over half a year. The main weakness in the experimental design is that one single site has used for the sampling, which likely reduces the generality of the results. The data analysis is also sound, although there are parts that lack the all the necessary details for repeatability, in particularly those related to the statistics (see detailed comments below). These details need to be provided before the paper can be published.
1. Response: Thanks for the comment. We have added a paragraph (Ln 354-360) in the Methods section to detail our statistical methods.

The introduction and results sections of the papers are well-written and clear. The discussion part is however extremely short and contains only a single paragraph. The conclusion is also very short and contains only a single sentence. Most of the findings described in the paper are therefore not discussed and put into a proper context, both regards to impact and existing research in the field. The authors need to significantly extend the discussion and conclusion before the paper can considered for publication.

2. Response: Thanks for the comments. Regarding to the issue of single-site sampling, the main purpose of this study is to analyze the temporal dynamics of PM microbiome in Beijing. In addition, this sampling site (Tsinghua Campus) is in the urban center of Beijing and is therefore suitable to provide some snapshots of Beijing’s PM microbiome dynamics. However, we will indeed expand our analyses in multiple sampling sites in other cities. Also thanks to the point of inadequate discussion, we have added two paragraphs in the Discussion to present the significance and relevance of our findings.

   Detailed comments
   Throughput the paper the authors adjust their p-values to avoid issues with multiple testing. There is however no details on how this was done. What method was used and how there the p-values adjusted? Provide information if adjustment was done per analysis (how many analyses in total) or global. Also, no adjustment seems to have been done for the large number of tests in Supplementary Figure S7 and S8.
3. Response: Thanks for the suggestion. Please see the Response No. 1, we have added details of statistical analyses in the Method section. The post-correction results of Fig. S7 and S8 are provided. As for Fig S8 I and J, there are no statistical differences after correction, thus we have removed the two panels.

Line 95-98: How were the correlations between taxonomic composition and meterological factors computed? The R2 values presented in Table S5 suggests a parametric model. However, the values are all extremely low indicating overall low correlations. Please provide the full details. Also, why were these p-values not adjusted for multiple testing?

4. Response: Thanks for the suggestion. We have added a description of statistical analysis in the Method section. We substituted “strong” with “significant”. The post-correction results were provided.

Line 116-119: How were the tests comparing the gene abundances between the three catalogues performed? Pair-wise comparisons? Please provide more information.

5. Response: Thanks for the point. For the comparison of three catalogs, Kruskal-Wallis rank-sum test was used. Additionally, we have added it in the revised manuscript.

Line 123: "Genes that potentially confer resistance to 9 different antibiotics were identified, which can be further divided into 22 different classes". What do the authors mean with "9 different antibiotics"? Types of antibiotics? And I assume that it was the genes that could be divided into 22 classes, not the antibiotics? Please rephrase.

6. Response: Thanks for the suggestion. Yes, we mean “types of antibiotic targets.” We have rephrased the sentence as follows: “Genes that potentially confer resistance to 35 different antibiotics were identified”.

Line 129: "MFS categories" -> "MFS transporter categories"

7. Response: Thanks for the suggestion. We have changed it in the revised manuscript.

Line 134: The low proportion of MGEs found next to ARGs shows that either 1) the authors failed to assemble the MGEs properly or 2) the authors ARG database contains a lot of non-ARG genes. Please elaborate on why this number is so low.

8. Response: Thanks for the suggestion. We have annotated the ARGs with CARD and identified MGEs with the ACLAME database. The proportion of MGEs was increased from 0.27% to 5.0%.

Line 152-153: This sentence is misleading. The only significance is between group I and II for PM10. There is no evidence of a general pattern.

9. Response: Thanks for the suggestion. We have rephrased the sentence as follows: “The number of detoxification genes was greater in AQI group II than in AQI group I”.

Line 191-193: ARGs are commonly present in all forms of bacterial communities. This study has not show that Beijing smog contains any ARGs of particular concern so this conclusion should be toned down.

10. Response: Thanks for the suggestion. We have removed this sentence.

Line 304-305: What does "the ARG database created using ResFAMs" mean? Also, how exactly were Uniprot used? It is well-known that public databases, such as Uniprot, contains a lot of incorrectly annotated resistance genes. The authors should instead use a well-established ARG database, such as ResFinder or CARD?

11. Response: Thanks for the suggestion. We have accordingly used the CARD to annotate the ARGs, and updated the relevant figures and tables. Additionally, we used ShortBRED[1] to quantify the abundance of the annotated ARGs from CARD. According to the instructions of this software, we identified the ShortBRED markers from the annotated antibiotic resistance proteins using the reference database of Swiss-Prot in Uniprot. Of note, Swiss-Prot is a curated protein database with a manual annotation that is more reliable than those derived from the automatic annotation. These approaches have been reported previously [2-4]. Additionally, changes were made accordingly in the revision, which contains more details for the bioinformatic methods.

[1] Kaminski J, Gibson MK, Franzosa EA, Segata N, Dantas G, Huttenhower C. High-specificity targeted functional profiling in microbial communities with ShortBRED. PLoS Comput Biol. 2015 Dec 18;11(12):e1004557.

[2] Pehrsson E C, Tsukayama P, Patel S, et al. Interconnected microbiomes and resistomes in low-income human habitats[J]. Nature, 2016, 533(7602): 212.

[3] Gibson M K, Wang B, Ahmadi S, et al. Developmental dynamics of the preterm infant gut microbiota and antibiotic resistome[J]. Nature microbiology, 2016, 1(4): 16024.

[4] Ju F, Beck K, Yin X, et al. Wastewater treatment plant resistomes are shaped by bacterial composition, genetic exchange, and upregulated expression in the effluent microbiomes[J]. The ISME journal, 2019, 13(2): 346.

Supplementary material: Figure S3 lacks resolution which makes it almost impossible to interpret.

12. Response: Thanks for the suggestion. We have improved the resolution.


    Reviewer #2: The manuscript of Qin at al is investigating the composition and functional potential of microbes residing on dust particles in an urban environment over time. The experimental design is sound and the analysis follows standard practices.  It is difficult to extrapolate how the temporal variation identified in Beijing would appear in different cities across the globe. Are those profiles signature-like for different cities or unified by an underlying external factor? A future study based on the same rigorous methodological approach would be able to shed light into such questions.
    I only have minor remarks concerning this work.
    1) It would be interesting to know the number of living microbes (non-viral) in the community. Is there literature known on dust particles plate colonies count or live-dead counts?
13. Response: Thanks for the suggestion. Yes, we are curious about this question too, as it is living microorganisms that directly influence human health. There are not so many studies into open-air living microorganisms while the majority focus on indoor environments like hospitals and operation rooms. One representative research reported that 26 groups of bacteria and fungi either of human or environmental origin were detected from outdoor air samples. 7 genera of fungi were found, mainly belongs to the genus *Aspergillus*. And bacteria occur in most environments; particularly in dusty, dirty places inhabited by human or other animals. Many of the species of bacteria isolated from the buildings are harmless and frequently include members of the genera *bacillus* and *micrococcus* and also *diphtheroids bacillus*.

Ref: Yassin, M. F., & Almouqatea, S. (2010). ASSESSMENT OF AIRBORNE BACTERIA AND FUNGI IN AN INDOOR AND OUTDOOR ENVIRONMENT. International Journal of Environmental Science and Technology, 7(3), 535-544.

2) What percent of the reads was not mapped by MetaPhlAn2?

14. Response: Thanks for the point. The percentage was 0.4%±0.5%. The references of MetaPhlAn2 were unique clade-specific marker genes, but not whole-genomes, so the mapped ratio was relatively low, such as the ratio of mapped reads for human gut samples was 2%-8%.

3) Negative controls are not mentioned. It would be interesting to see what the profile of a filter remaining in the sealed bag for a day and then followed the same treatment would look like.

15. Response: Thanks for the point. We used negative controls as detailed in the follows. A membrane was cut into quarters, which were sterilized. The pieces were not used to collect particles but were nevertheless extracted according to the same protocol as the normal sampling membranes. The concentration of negative control was measured by Qubit, and the readings were undetectable three times and were recorded as N/A. We have now specified this in the revision (Line 247-249).
4) The sequence data for the project are not retrievable yet. Only the project is registered.
16. Response: Thanks for the suggestion. The sequence data has been released.

5) Line102 conincided should be coincided.

17. Response: Thanks for the suggestion. We have corrected it.

6) Line 359 beteween should be between

18. Response: Thanks for spotting the typo. We have corrected it.

7) Line 368 beteween should be between

19. Response: Thanks for spotting the typo. We have corrected it.

**Second round of review**

**Reviewer 1**

The authors have addressed almost all my concerns. I only one very minor comment on the new paragraph describing the statistical analysis.

Line 353: "The multiple testing of all the p-values in each test was performed using Benjamini and
Hochberg false discovery rate (FDR) controlling procedure (p.adjust function in R)." should be changed to "Adjustment for multiple testing was done using Benjamini and Hochberg false discovery rate (FDR) controlling procedure (p.adjust function in R)"

(The adjustment is done per testing situation, not individual test, but this result in a test-specific q-value which is used to control the FDR).

**Reviewer 2**

Thanks for the consideration of my comments.
